# Advances in plant gene-targeted and functional markers: a review

**DOI:** 10.1186/1746-4811-9-6

**Published:** 2013-02-13

**Authors:** Péter Poczai, Ildikó Varga, Maarja Laos, András Cseh, Neil Bell, Jari PT Valkonen, Jaakko Hyvönen

**Affiliations:** 1 Plant Biology, Department of Biosciences, University of Helsinki, PO Box 65, 00014, Helsinki, FIN, Finland; 2 Institute of Biotechnology, University of Helsinki, PO Box 65, 00014, Helsinki, FIN, Finland; 3 Agricultural Institute, Centre of Agricultural Research, Hungarian Academy of Sciences, PO Box 19, H-2462, Martonvásár, Hungary; 4 Botanical Museum, University of Helsinki, PO Box 7, 00014, Helsinki, FIN, Finland; 5 Department of Agricultural Sciences, University of Helsinki, PO Box 27, 00014, Helsinki, FIN, Finland

**Keywords:** DNA fingerprinting, Functional markers, Gene targeting, Genomic database, Molecular markers

## Abstract

Public genomic databases have provided new directions for molecular marker development and initiated a shift in the types of PCR-based techniques commonly used in plant science. Alongside commonly used arbitrarily amplified DNA markers, other methods have been developed. Targeted fingerprinting marker techniques are based on the well-established practices of arbitrarily amplified DNA methods, but employ novel methodological innovations such as the incorporation of gene or promoter elements in the primers. These markers provide good reproducibility and increased resolution by the concurrent incidence of dominant and co-dominant bands. Despite their promising features, these semi-random markers suffer from possible problems of collision and non-homology analogous to those found with randomly generated fingerprints. Transposable elements, present in abundance in plant genomes, may also be used to generate fingerprints. These markers provide increased genomic coverage by utilizing specific targeted sites and produce bands that mostly seem to be homologous. The biggest drawback with most of these techniques is that prior genomic information about retrotransposons is needed for primer design, prohibiting universal applications. Another class of recently developed methods exploits length polymorphism present in arrays of multi-copy gene families such as cytochrome P450 and *β*-tubulin genes to provide cross-species amplification and transferability. A specific class of marker makes use of common features of plant resistance genes to generate bands linked to a given phenotype, or to reveal genetic diversity. Conserved DNA-based strategies have limited genome coverage and may fail to reveal genetic diversity, while resistance genes may be under specific evolutionary selection. Markers may also be generated from functional and/or transcribed regions of the genome using different gene-targeting approaches coupled with the use of RNA information. Such techniques have the potential to generate phenotypically linked functional markers, especially when fingerprints are generated from the transcribed or expressed region of the genome. It is to be expected that these recently developed techniques will generate larger datasets, but their shortcomings should also be acknowledged and carefully investigated.

## Introduction

In recent years, many promising new alternative molecular marker techniques have been developed in plant genetics, largely due to rapid growth in genomic research initiating a trend away from random DNA markers towards gene-targeted functional markers [1]. Due to the rapid expanse of several public genomic databases, the development of functional markers, which are located in or near candidate genes of interest, has become relatively simple [2]. These markers play a key role in, for example, studies of genetic variability and diversity, the construction of linkage maps, and tracking individuals or lines carrying particular genes [3]. They can be used to select and pair parental genotypes or to eliminate linkage drag in back-crossing, and also to select traits that are difficult to measure using phenotypic assays [4]. Molecular markers have many other applications, including in phylogenetics and systematics, conservation biology, molecular ecology and developmental biology, as well as numerous uses in forensics, disease testing and paternity assessment. A historical example can perhaps illustrate how important the specific nature of a marker can be. Between 1816 and 1820, sheep breeders in Brno were debating the association of wool traits (color, fitness, density, etc.) and how to effectively combine useful traits in progenies [5]. Imre (Emmerich) Festetics (1764–1847), a Hungarian noble from Keszthely (Georgikon) was active in these discussions and performed a number of crossing experiments [6]. Based on his results he formulated some rules of heredity and was the first to refer to such principles as “Genetic laws of Nature” (“*Die genetische Gesätze der Natur*”), in a series of papers about inbreeding published between 1819 and 1822 that preceded Gregor Mendel by a generation [7]. He used the term "genetic" 80 years before Johannsen and Bateson. Unfortunately, the markers of choice were traits subject to polygene inheritance such as wool density and length, and conclusions similar to Mendel’s would have required precise techniques and solid statistical methods, such as those known today as quantitative trait loci (QTL) mapping. However, Festetics summarized his results in the form of four “genetic laws”, pointing out that race traits in sheep are intrinsic and can be “concentrated” by inbreeding. He also linked heredity (*Vererbung*) with health and vigor independent of external factors, and stated that the traits of grandparents may reappear in later generations, while animals with similar traits may have divergent offspring. Although the emergence of genetics was undoubtedly delayed, there is no evidence that Mendel ever read or cited the work of Festetics, which was in the library in Brno. Later Mendel, fortunately chose to investigate characters (markers) in peas (*Pisum* L.) which are monogenic, thus allowing him to clearly postulate the laws of inheritance. An ideal marker should be polymorphic, independent, and reliable, providing sufficient resolution relatively easily, quickly and with fairly low costs. Depending on the nature of the study many other characteristics may also be important. In plant breeding it is essential to know how a marker is linked to a desired trait (phenotype), but this is not relevant for genetic diversity or phylogenetic studies. On the other hand, phylogenetic studies greatly benefit from molecular techniques requiring relatively small amounts of DNA or organismal material, as in many cases tissue for such studies is very restricted. However, this is largely irrelevant in plant breeding programs, where a large amount of fresh plant material is almost always available.

The emergence of most biochemical marker systems has closely followed advances in biochemistry and molecular biology [3,8]. Techniques such as isozyme analysis were pioneering methods in molecular marker studies [9]. The pitfalls and shortcomings of such data were soon recognized (for example distortion due to co-dominant inheritance, environmental and genetic post-translational modifications, and problems with polyploid duplication), leading to the development of DNA-based markers [10]. The dominance of techniques based on restriction fragment length polymorphism (RFLP) ended with the introduction of the polymerase chain reaction (PCR; [11]), which resulted in a widely applied categorization of molecular markers as either non-PCR based or PCR based, further subdivided into single and multi-locus methods. Genome sequencing projects have been influenced both by the discovery of gene structures and by single nucleotide polymorphisms (SNPs). SNP genotyping aims to reduce costs and facilitate high throughput assessment by using plates of 384 reactions, or by applying multiple loadings of gels and automatic sequencers [12]. These techniques, coupled with next generation sequencing technologies (NGS), have rapidly resulted in ultra-high-throughput, low-cost assays for a variety of new marker technologies. SNP technologies have been reviewed by Gupta et al. [12] and detailed by Henry [13], and are beyond the scope of this review. Here we mostly concentrate on advances made in multi-locus technologies for plant genotyping. Basic techniques such as AFLP, ISSR and RAPD, as well as other non-PCR based approaches such as RFLP will not be discussed here in detail. Instead, we provide an overview of recent progress made in these methods and highlight improvements relevant to gene-targeted and functional markers. Basic single-locus markers, e.g., microsatellites (SSRs), as well as advanced techniques in this group will also not be discussed, despite their popularity and usefulness. We review developments in PCR-based multi-locus techniques that either incorporate modifications to existing methods or rely on new principles, and belong to the class of gene-targeted and/or functional markers. We also summarize briefly their advantages and potential drawbacks and propose a classification for existing technologies.

## Arbitrarily amplified DNA markers (AADs)

**Figure 1:**
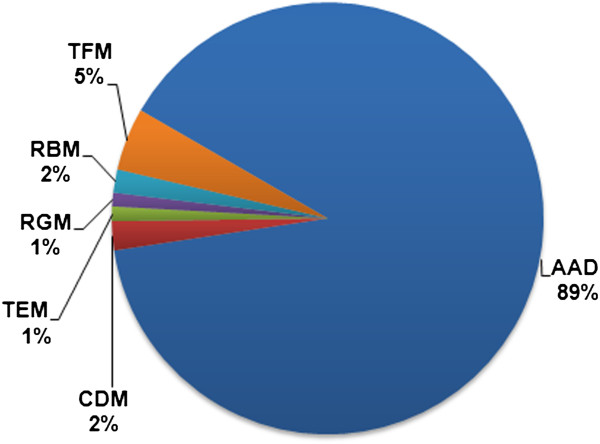
**Percentages of studies utilizing different types of molecular markers.** The chart is based on an informal literature search performed with Google Scholar on 22.08.2012 resulting in 1032570 hits. Abbreviations are according to acronyms found in the text: **AAD** – Arbitrarily amplified DNA markers, including AFLP, ISSR, RAPD, and other modified but similar methods mentioned in the text; **CDM** – conserved DNA based markers, including CDDP, PBA, TBP, ITP (all modified methods are cited in the text); **TEM** – transposable element based markers including IRAP, REMAP, ISAP, iPBS and SSAP. **RGM** – resistance-gene based markers (RGAP), NBS-profiling; **RBM** – RNA-based markers, iSNAP, EST- and cDNA- based markers; **TFM** – targeted fingerprinting markers (DALP, PAAP, SRAP, TRAP, CoRAP and SCoT).

Before describing recent developments, some aspects of the first PCR-based methods must be discussed. Techniques in this group use genetic markers that occur at multiple sites throughout the genome, thus banding patterns are a product of amplification from multiple priming sites. By sampling multiple loci simultaneously they can be useful for solving a number of problems that may be hard to address using single-locus methods such as those associated with introgression and hybridization studies. The major advantage of technologies based on arbitrarily amplified markers is that there is no need for any *a priori* sequence information from the analyzed organism. Most dominant markers are generated randomly over the whole genome, sampling multiple loci. These methods are technically simple, fairly cheap and generate a relatively large number of markers per sample. Many types of multi-locus methods are well known and include random amplified polymorphic DNA (RAPD, [14,15]), amplified fragment length polymorphism (AFLP, [16]), inter-sample sequence repeats (ISSR, [17]) and a few alternative techniques involving some modifications of these [18-23]. They are still used and have many applications [24-26]. Collectively they have been referred to as arbitrarily amplified DNA markers (AADs; excluding single-locus techniques such as microsatellites, or SSRs). During the last two decades thousands of studies have utilized AADs in plant science for various purposes [27]. We performed an informal search using Google Scholar to obtain a rough estimate of how many studies have utilized AAD markers and compared the values to those obtained for the other marker types reviewed here. The percentages presented in our pie chart (Figure 1) should be interpreted with caution, but it seems that AADs are still popular techniques.

Many studies have highlighted weaknesses [27-29] of these techniques but without proposing alternative approaches. These shortcomings include: i) co-migration of fragments of same size originating from independent loci among different analyzed samples; ii) co-migration of bands that are paralogous rather than orthologous; iii) nested priming, leading to amplicons derived from overlapping fragments; iv) heteroduplex formation, where products are also generated from alternate allelic sequences and/or from similar duplicated loci; v) collision, where two or more equally sized but different fragments occur within a single lane; vi) non-independence, where a band is counted more than once due to co-dominance or nested priming; vii) artifactual segregation distortions, caused by mistaken scoring of loci, undetected co-dominance, or poor gel resolution [27-29]. Besides these common problems each method has its own specific drawbacks not detailed here (see [27,28]). Some techniques have been thoroughly reviewed [30,31], had their technological features investigated in detail [32,33], or, after a few years of neglect, have been resurrected [34]. Nonetheless, the shortcomings listed above still apply. Does this mean that the reintroduction of a technique after a period of disuse can lead to it becoming more popular, or that after a few years its drawbacks are forgotten? It is difficult to say, but in any case it seems that if used cautiously [35] with appropriate restrictions on sampling strategies [36] and careful design of experiments, useful information can still be achieved with AADs providing that the limitations of the techniques are kept in mind. Exploring the limits of AADs has certainly resulted in them being better exploited, while it has also initiated a shift towards more restricted applications. Although historically these systems have had wide ranging applications, their use is becoming increasingly restricted to particular scientific fields in which they are specifically warranted, while they are being superseded by other methods in the areas in which they fail. One example is the use of AAD techniques in phylogenetics. Recent studies have shown that AAD markers can be useful in addressing phylogenetic questions in recently radiated and closely related species [37,38]. The main argument against the use of AADs in phylogenetics is the claim that they merely provide homoplastic “noise”. However, homoplasy may be less of a problem for AAD markers in very closely related species with a similar genomic organization than it is in distantly related species, and hence these problems may be largely a result of attempts to use such markers at inappropriate phylogenetic levels [39,40]. There also seem to be fewer problems with organisms that reproduce clonally or in alternative asexual ways, e.g., some microscopic fungi. Some journals require authors to justify the use of AADs, and thus promote their appropriate use. Improvements have also been made in resolving banding patterns by fluorescent labeling of primers [41-43]. This has resulted in improved techniques such as FluoMEP [44]. Automated fragment analysis [37] is also increasingly used together with other technological or methodological advances such as different scoring protocols [45,46] or statistical corrections [29,47,48]. Software specifically designed for band scoring [49] has also been developed. The critical evaluation of the shortcomings of AADs in the recent studies described above provides a good example to follow when considering other marker systems developed subsequently.

## Gene-targeted and functional markers (GTMs and FMs)

The major difference between anonymous dominant markers (AADs) and functional or gene targeted markers is the way they are generated. A molecular marker can be derived from any stretch of DNA showing polymorphism and tagged by a primer of variable length. However, in many cases the utility of such neutral markers can be negated by a simple recombination, limiting the use of arbitrarily amplified DNA markers [50]. In other words, non-targeted amplicons may either belong to the transcribed or non-transcribed region of the genome; they have been developed without knowledge of their function. Structural and functional genomic research projects in several plant species, e.g., potato, *Solanum tuberosum* L. [51]; soybean, *Glycine max* (L.) Merr. [52]; ryegrass, *Lolium perenne* L. [53] and maize, *Zea mays* L. [54] have resulted in additional information allowing systematic development of targeted markers derived from polymorphic sites within genes that affect phenotypic trait variation [2]. It is important to make a distinction between gene-targeted markers (GTM) and functional markers (FM), because not every GTM is involved in phenotypic trait variation and thus may not become functional. Gene-targeted markers can also tag untranslated regions of expressed sequence tags [55,56]. Following the definition proposed by Andersen and Lübberstedt, [2] functional markers are derived from polymorphic sequences, and are more likely to be involved in phenotypic trait variation. Based on this conceptual framework, the marker systems discussed below are all (gene)-targeted markers, which have the potential to become functional. Recently many new marker systems of this type have been developed (Table 1).

**Table 1:** Summary table of marker systems and groups

**Group**	**Marker system**	**Principle in a nutshell**	** References**
(1) Conserved DNA and gene family based markers (CDMs)	(1.1) CDDP	Conserved plant genes are targeted with short universal or degenerate primers to reveal length polymorphism. Use of primer combinations is also possible.	Collard and Mackill [57]
	(1.2) PBA	Universal primers target the exon-intron junction sites of cytochrome (cyt) P450 mono-oxygenases. Polymorphism is revealed based on the random distribution of gene family members.	Yamanaka et al. [58]
	(1.3) TBP	Single degenerate primer pairs anneal to the conserved parts of the β-tubulin exons and amplify intercalated introns from different tubulin isotypes.	Bardini et al. [59]; Breviario et al. [60]; Galasso et al. [61]
	(1.4) ITP	Intron regions of choice are amplified by exon flanking primers revealing polymorphism.	Weining and Langridge [62]
(2) Transposable element based markers (TEMs)	(2.1) IRAP	Amplification of internal sequences between two retrotransposon repeats with primers annealing to LTR motifs.	Kalendar et al.[63]
	(2.2) REMAP	An LTR specific primer and an ISSR primer are used to detect polymorphism.	Kalendar et al. [63]
	(2.3) ISAP	Primers designed in various positions within SINE elements are used to amplify adjacent genomic regions.	Seibt et al. [64]
	(2.4) iPBS	Primers anneal to PBS regions of head-to-head oriented LTR retrotransposons. The amplified products contain LTR regions and intervening genomic regions.	Kalendar et al. [65]
	(2.5) SSAP	DNA is digested with restriction enzymes. Adapters are ligated to restriction sites, and amplification is performed with LTR specific and adapter specific primers containing selective nucleotides.	Waugh et al. [66]
(3) Resistance-gene based markers (RGMs)	(3.1) RGAP	Resistance-gene based analogue fingerprints are generated with degenerate specific primers or primer pairs, designed to match conserved regions of R-genes.	Leister et al. [67]
	(3.2) NBS-profiling	Genomic DNA is digested with restriction enzymes after the ligation of adapters. Specific fingerprints are generated from resistance gene regions with adapter specific and R-gene specific primers.	Linden et al. [68]
(4) RNA-based markers (RBMs)	(4.1) iSNAP	Primers are designed from small RNAs and flanking regions to generate polymorphic banding patterns.	Gui et al. [69]
	(4.2) cDNA-AFLP	An AFLP analysis is carried out using cDNA as a starting pool, with several modifications existing for fine-tuning.	Bachem et al. [70]
	(4.3) cDNA-RFLP	cDNA is used for probes in RFLP analysis.	Bryan et al. [71]
	(4.4) EST-SSR	EST databases are mined *in silico* to locate SSRs and design primers to match genetic microsatellites.	Kantety et al. [72]
(5) Targeted fingerprinting markers (TFMs)	(5.1) DALP	The common M13 sequencing primer is paired with a forward primer containing the −40 USP core and 3’ selective nucleotides to generate fingerprints.	Desmarais et al. [73]
	(5.2) PAAP	Degenerate regions annealing to plant promoter regions are added to short oligonucleotides to detect polymorphism.	Pang et al. [74]
	(5.3) SRAP	Primers contain a random 5’ filter, a core sequence (AATT or CCGG) and three variable nucleotides at their 3’. Amplification follows a two step procedure where first mismatches are allowed at a lower temperature to generate a starting pool for subsequent higher temperature amplification.	Li and Quiros [75]
	(5.4) TRAP	An arbitrary SRAP primer is paired with a fixed primer designed from ESTs.	Hu and Vick [76]
	(5.5) CoRAP	Arbitrary primers are designed from ESTs as in TRAP, but the fixed primer contains a different core (CACGC), as in SRAP. This sequence is often found in plant introns.	Wang et al. [77]
	(5.6) SCoT	ATG start codons are incorporated into random primers to generate polymorphic fragments from the genome. Primers can be used alone or in combination.	Collard and Mackill [78]

### Conserved DNA and gene family based markers (CDMs)

Depending on the purpose of the study, functional markers, instead of non-functional ones, may be preferred. However, non-coding DNA also has many applications, e.g., SSR based cultivar identification and the use of non-coding chloroplast DNA in systematics [79]. When functionality and the resolution provided by slowly evolving DNA regions or fast evolution of SSRs is a problem, conserved DNA or gene family based markers may be good choices. Markers belonging to this family can be regarded as a special group of gene-targeted markers (GTMs), which utilize length polymorphisms of exon-intron structures in different widely distributed and common plant genes or gene families. Such techniques yield multi-locus markers generated from randomly distributed members of a targeted gene (family), varying in length and with a high potential of being functionally related to a given phenotype. Designing conserved DNA based primers without prior knowledge of the whole genome is essential to combine the advantageous features of multi-locus profile generation with functionality. Plant genomes include many gene-families that can be targeted with methods such as the ones described below. Only a few attempts have been made to develop new marker systems belonging to this group, and it is clear that there is a huge unutilized potential for marker development provided by conserved DNA regions and different plant gene-families.

#### Conserved DNA-derived polymorphism (CDDP)

Conserved genes, or, ideally, sequences of gene families present in multiple copies in the plant genome, can be amplified by short primers as described by Collard and Mackill [57]. Across functional domains of well-characterized plant genes these short tags can then generate informative banding patterns that have many uses, e.g., germplasm genetic diversity assessment, or mapping and trait association studies. In general, specific primers are designed in such a way that they anneal to conserved parts of these common functional genes, e.g., homeobox (KNOX) or auxin-binding protein (ABP1) coding genes, with the aim of generating polymorphic banding patterns that are detected on agarose gels. Resolution depends on the user and on the laboratory facilities available; primers may be fluorescently labeled for automation. Given the relatively large number of conserved gene regions and gene families in plant genomes, any region can be tagged using this technique. Collard and Mackill [57] describe a set of primers that target well characterized plant genes involved in responses to abiotic and biotic stress or plant development, but with further bioinformatic work this could be considerably extended. CDDP can easily generate functional markers (FM) related to a given plant phenotype. Conserved DNA regions sharing the same priming site, but differing in their genomic distribution, can yield a large number of easily detectable length polymorphisms. The technique is based on single long primer amplification with a high annealing temperature, which improves reproducibility. However, there have also been attempts to combine primers in CDDP reactions to amplify polymorphic regions representing DNA stretches between two identical or very similar conserved primer binding sites [80]. The reproducibility of the technique has proved to be high compared to traditional AADs. However, some primer problems can occur, suggesting that primer length and high annealing temperatures may not ensure complete reproducibility. This indicates that scoring of banding patterns should be based on replicates, and results should be treated cautiously.

#### Cytochrome P450 based analogues (PBA)

**Figure 2:**
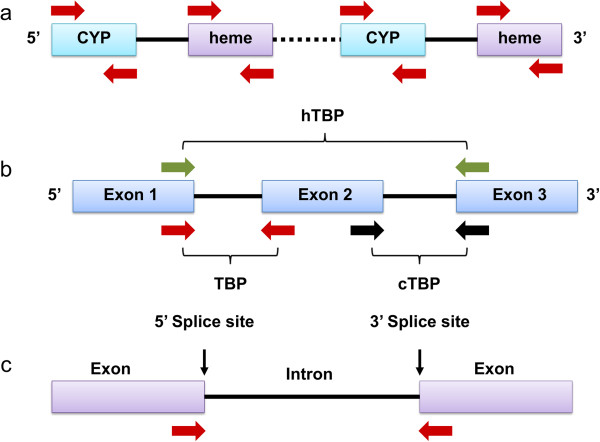
**Schematic representation of conserved DNA and gene family based markers.** Color boxes represent exons and solid black lines introns, while intergenic regions are indicated by dashed lines. Red, green and black arrows are primers used for amplification in each technique. **a)** The cytochrome P450 Based Analogue (PBA) marker system is based on the amplification of Cyt P450 regions in plants with universal primers designed in CYP or heme-binding sites. **b)** Representation of the transcribed region of a typical plant *β*-tubulin gene, showing specific amplification with TBP (red arrows), cTBP (black arrows) and hTBP (green arrows). **c)** Outline of intron-targeting markers with primers flanking the exon regions. A similar system can be applied to methods using conserved DNA-Derived Polymorphism (CDDP).

This technique, developed by Yamanaka et al., [58] employs cytochrome P450 based analog (PBA) markers and also uses conserved and widely distributed plant gene families to detect polymorphism (Figure 2). The targeted specific regions are coding cytochrome (Cyt) P450 mono-oxygenases, which are highly abundant in plants, fungi and other microorganisms, as well as in animals [81]. In embryophytes they play important roles in oxidative detoxification and in the biosynthesis of secondary metabolites [82]. It has been reported that the sequence diversity of P450 gene-analogues in plants is useful for studies at both the functional and genome-wide scales [83]. Data mining of the genome sequence of the model plant *Arabidopsis thaliana* (L.) Heynh. has resulted in the development of a number of primer-sets derived from Cyt P450 genes, which have the potential to be used in diverse plant species lacking other relevant genetic markers [58]. Since Cyt P450 genes are widely distributed within the plant genome they can be utilized to create polymorphic fingerprints to characterize genetic diversity within and among populations of a wide variety of plant species. The genomic annotation of *Arabidopsis* revealed that out of the ~ 29,000 genes in the genome, nearly 0.9% (272 genes and 26 pseudogenes) are putative Cyt P450 genes [84]. This indicates that these genes are very diverse, providing the opportunity for them to be utilized in diversity assessment. In the method developed by Yamanaka et al. [58] universal primer pairs, designed to anneal to specific conserved exon regions of Cyt P450 genes, are arbitrarily paired. Forward and reverse primers flanking the intron regions are then used to initiate PCR amplification. Based on the random distribution of Cyt P450 genes in the genome, the resulting banding patterns will reflect polymorphism based on the variation found across the targeted (pseudo)genes. Cross-species amplification and transferability of PBAs was reported and verified for 52 different species from 28 families [58].

#### Tubulin based polymorphism (TBP)

Tubulin synthesis in plants is based on the *α*- and *β*-tubulin gene families, these genes coding for the two distinct polypeptide building blocks that form microtubules. These elements have multiple roles in the cell as they are essential for cell division, vesicular transport, signal propagation, cell wall deposition and many more activities [85]. Plant *β*-tubulin genes have typical conserved sequences with two intercalated introns in fixed positions, the only known exception being in maize, where the second intron is lost [86]. Moreover, these introns have been conserved throughout their evolution, and are found in the same well-defined positions within their respective genomic sequences in organisms as diverse as yeasts and flowering plants [86-88]. The introns have a role in the control of tubulin gene expression in plants, as reviewed by Breviario [88]. The polymorphism of these regions also provides a good basis for genetic diversity assessment, identification of different plant varieties for breeding purposes, and even the investigation of eukaryotic evolution [89]. Based on these features Bardini et al. [59] developed an assay, Tubulin Based Polymorphism (TBP), to reveal length polymorphisms present in the introns (Figure 2). For this rapid technique they designed a single degenerate primer pair annealing to the conserved parts of the *β*-tubulin exon after the 132 amino acid codons. These specific primers flanking the intron splicing site enable the amplification of the first intron of different *β*-tubulin isotypes, revealing specific fingerprints. The resulting banding patterns are separated on polyacrylamide gels and show variation comparable to SSR markers in species of *Brassica* L., *Coffea* L. and *Lotus* L. Further modification of the technique was proposed by Breviario et al., [60] and termed combinatorial TBP (cTBP). Here the original primer set for intron I is modified to enhance reliability and new primers are designed to flank the second *β*-tubulin gene intron. Anticipated results of TBP fingerprinting are shown in Additional file 1. Galasso et al. [61] introduced new primers for amplification of the entire *β*-tubulin region, containing the partial exons 1 and 3 and the full sequences of introns I and II, as well as exon 2. They named this variant h-TBP (horse-TBP). By cloning the resulting banding patterns from *Camelina sativa* (L.) Crantz, it was revealed that amplification was achieved from the corresponding sites with considerable variation preserved in the introns of more than 30 different members of the *β*-tubulin gene family.

#### Intron-targeting polymorphism (ITP)

Introns have long been considered as a source of polymorphism due to their moderate sequence evolution, which is presumed to take place under minimal constraints in a fashion consistent with the neutral theory of sequence evolution [90]. Recent reports have shown intron length polymorphism to be a convenient and reliable source of information with high interspecies transferability. Introns can be exploited for the construction of genetic maps, because they directly reflect variation occurring within genes [91]. Insertion-deletions (indels) of introns are becoming important genetic markers for many plant taxa [92]. The basic approach is termed intron-targeting (IT) and uses intron splice junction (ISJ) primers, as described by Weining and Langridge [62]. In this initial study intron length polymorphism in the *α*-amylase gene family was used. Further modifications of the method have been presented by many authors [93-98]. However, the lack of large plant genomic databases has halted the development of primers and full exploitation of the method. The technique itself has been referred to under many names, including exon-primed intron-crossing (EPIC) PCR [96], conserved-intron scanning primers (CISP; [99]), intron-flanking primers [100], potential intron polymorphism (PIP; [101]) and PCR-based landmark unique gene (PLUG) markers [102]. There are minor differences between these techniques, e.g., in the source used to obtain the primers, the resolution method after PCR amplification, and the intron regions amplified by the primers. However, they undoubtedly all rely on the same fundamental technique, here referred to as intron-targeting polymorphism (ITP) as proposed by Weining and Langridge [62]. Intron-targeting markers (Figure 2) can originate from either multiple or single loci depending on the features of the targeted regions. In this respect, the techniques discussed above can be regarded as specific types of intron-targeting which are exclusively used for a given gene family or conserved DNA region. Another important feature of intron-targeting is that primers can be generated from genomic or EST databases from various regions of the genome. These primers may correspond to intron length polymorphic sites of any gene or gene family, and are generated from conserved exon sequences flanking the introns in order to exploit intronic polymorphism discovery rates and to allow cross-species applications and transferability. The close proximity of introns to exons makes them well suited for the detection of length polymorphism in their structure that can be utilized for various purposes [98]. The successful transferability and cross-species amplification capacity of IT markers depends on the conservation of exon-intron junctions and gene structures across related genomes in different taxa. If the shared syntenies of the targeted genes as well as their sequence features are relatively conserved, primers can be transferred easily between taxa. This phenomenon is valuable for generating functional markers directly related to gene regions and facilitating the discovery of specific markers linked to a given phenotype (Additional file 2). It is also possible to tag specific genes related to environmental factors that could have useful applications, for example in molecular ecology. This is because IT uses primers based on allele sequences of functionally characterized genes, and thus specific banding patterns corresponding to plant phenotypes can be identified [103,104]. However, development of such markers depends on the availability of genomic databases with several target sequences for IT markers. Functional gene characterization might be a limiting factor, since it is not possible to establish functions for all genes. The crucial question is whether useful allelic variation can be identified for all genes of (for example) ecological relevance in the targeted organism.

## Utility and limitations of conserved DNA based markers

In the application of molecular markers, selection criteria include the speed and ease of processing information, cost-efficiency, reproducibility, and the quantity and type of genetic information that will be obtained [105]. AAD markers undoubtedly meet these criteria, but as discussed above, many concerns have been raised about their use (see [27,28]). Some studies have suggested that amplification of AADs from the genome is biased depending on the applied technique [32]. It is known that some AADs, such AFLPs, tend to be clustered around the centromeric regions in plants [106,107]. It has also been observed that some clusters occur only within particular chromosomal areas due to the enrichment of AFLP markers in certain regions, [108] and in several species this indicates recombination suppression [109,110]. The results of studies based on conserved DNA and gene family related markers [57,60,98,111] reveal that the obtained fragments show polymorphism in a wide range of plant species, suggesting that these markers could be useful tools for within or among population genetic diversity assessment (Table 2). An extensive list of relevant information with useful references to the application areas of CDMs can be found in Additional file 3.

**Table 2:** Comparison of various aspects of gene-targeted and functional marker techniques

	**Conserved DNA and gene family based markers (CDMs)**				**Transposable element based markers (TEMs)**			
	**CDDP**	**PBA**	**TBP**	**ITP**	**IRAP/REMAP**	**ISAP**	**iPBS**	**SSAP**
Abundance	Medium, may depend on targeted genes	High	Medium	Low, may depend on targeted genes	High	High	High	High
Reproducibility	High	High	High	High	Medium	High	High	High
Polymorphism	Medium	High	High	Medium	Medium	Medium	High	High
Prior sequence information	Yes	No	No	Yes	Yes	Yes	No	Yes
Visualization	Agarose gel electrophoresis	Agarose gel electrophoresis	Agarose gel electrophoresis or silver stained PAGE	Agarose gel electrophoresis sometimes with high resolution	Agarose gel electrophoresis	Agarose gel electrophoresis	Agarose gel electrophoresis	Silver stained PAGE
Specificity	Not reported	High	High	High	High	High	High	High
Size of bands	200-1,500 bp	100-1,500 bp	500-2,000 bp	50-800 bp	100-5,000 bp (up to 10 kbp)	250-2,500 bp	100-5,000 bp	50-500 bp
Homoplasy	High	High	Low	Low	Medium	Not reported	Low	Low
Reaction artifacts								
i. Uniparental bands	Not reported	Not reported	No	No	No	Not reported	No	No
ii. Heteroduplexes	Not reported, but may occur	Not reported, but may occur	Not reported, but may occur	Yes	No	No	No	No
iii. Nested priming	Not reported	Not reported, but may occur	No	No	May occur	Not reported	Not reported	May occur
iv. Other	Amplicons may be generated from pseudogene loci				Inconsistencies in bands associated with TE activity	No	No	Inconsistencies in bands associated with TE activity

### Size range and genome coverage

The size of the conserved intervening sequences amplified by the designed primers can be highly variable. This might be useful for classification at lower taxonomic levels, either alone or in conjunction with other multi-locus or sequence based methods. Conserved DNA markers can help to characterize the diversity of different species and detect inter- and intraspecies variation [58,59]. Their inheritance follows Mendelian rules, making them suitable for population studies. The individual members of plant gene families are often closely arranged in the genome, providing almost a single-locus target, [112] although this varies according to the family. Conserved DNA markers combine reliability and reproducibility with easy access to the generated raw data. A further advantage of the markers is that no prior information on specific sequences is required once the primers have been designed based on the available data. The universal primers are usually easily transferable between diverse taxonomic groups due to the conserved nature of the targeted genes. Banding patterns are based on length polymorphism, which requires no further laboratory treatments. However, novel primer design may become problematic if genomic annotations of conserved sequences are missing. It has also been shown that conserved DNA based markers are able to discriminate between different species, that the number of amplified bands correlates well with ploidy levels [61], and that banding patterns can reflect rearrangements of polyploid genomes. Such experiments have also been performed using AAD markers, with various results [113,114]. The development of such tools for estimating genetic diversity based on functional segments of conserved DNA sequences can contribute to bridging the gap between genotypes and phenotypes.

### Locus specificity

Some results using conserved DNA based primers have shown that PCR products obtained from different plant species may not only amplify the targeted specific genes, but also multiple analogues of the investigated gene family [58]. The extent of this problem seems to vary between different techniques. Galasso et al. [61] cloned a number of TBP fragments and found no amplicons from analogous sites. Cernák et al. [103] proved the amplification of targeted introns from the corresponding gene using simple restriction digestion. For PBA markers, Yamanaka et al. [58] found that not all fragments were associated with plant P450 genes, but at least some of the amplified products were plant P450-associated. Unfortunately, no such studies are available for CDDP markers. These results highlight that non-specific fragments tend to appear when larger gene-families are targeted (e.g., PBAs), but remain insignificant or unnoticed in conserved genes with few copies. This phenomenon is problematic in studies where homology of the bands is essential (e.g., in systematics and evolution). However, other fragments related to the targeted or expected gene loci might still be useful for functional marker studies (e.g., breeding and genetic mapping), as well as for genetic diversity assessment.

### Limitations to uncovering of genetic variation

Another drawback of conserved DNA based markers is that they can fail to identify variation in highly inbred species, even if the techniques used rely on different genes or gene families. This applies to any species that has experienced a severe genetic bottleneck followed by range expansion and rapid dispersal, or to cultivar groups based on limited genetic diversity. One additional deficiency of these techniques is that they can only detect a limited set of markers exactly corresponding to the targeted gene(s) or associated regions. This might be attributed to the fact that conserved gene regions tend to have less preserved sequence variation. However, this can be avoided to some extent with additional improvements such as choosing more variable regions like PBAs, although this would be accompanied by the generation of non-specific products as mentioned previously. It is also possible to choose genes with less length variation and genomic distribution, but with preserved exon-intron structures.

### Amplification artifacts

**Figure 3:**
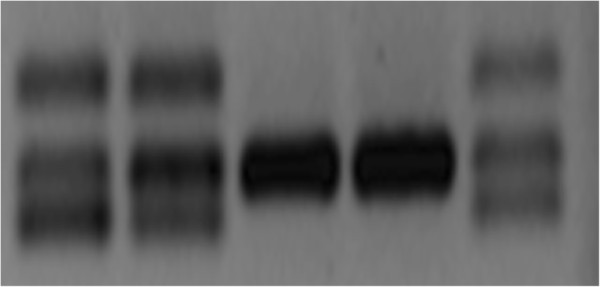
**Heteroduplex formed by different DNA strands from homologous sites.** Additional band originating from different alleles of the *Cat*-*In*2 locus, linked to the *Ry*_*sto*_ gene in *Solanum stoloniferum* Schltdl. The heteroduplex is the first band in the upper row. The artifact was investigated with single-strand conformation polymorphism (SSCP) analysis (not shown), where this band was not detected due to it being composed of two different strands from the lower two fragments. Photo kindly provided by István Cernák.

Depending on the gene region, banding patterns can be highly variable, and it might be difficult to predict the exact size of PCR products for gene families producing multiple products. These can result from pseudogene loci, or from PCR errors generating artifacts. However, polymorphic bands are reproducible under similar reaction conditions. The PCR conditions should be carefully optimized, because non-specific amplification products, such as heteroduplexes, may occur. Conserved DNA and gene family based marker polymorphism results from insertions/deletions in the amplified fragments, representing different alleles of the targeted gene. Therefore, amplicons can form heteroduplex artifacts, where a double-stranded product is generated from single complementary strands derived from alternate allelic sequences of the targeted gene. An example is shown in Figure 3 for the intron-targeting method. Conserved regions allow easy transition from multi-locus to single-locus applications where further SNPs require additional downstream processing. However, even with this shift, conserved DNA region based markers might reach their limits if used to study the phylogeny of inbred taxa. Nonetheless they may be useful for (molecular) ecological studies aiming to characterize diversity in different ecological niches and geographical areas.

### Transposable element based markers (TEMs)

**Figure 4:**
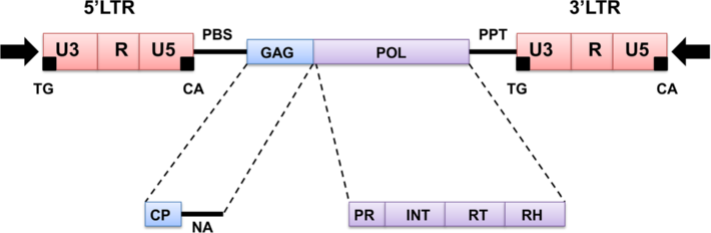
**Structure of a plant Ty1-*copia *retrotransposon, which contains two long terminal repeat (LTR) elements at either end (red boxes) surrounded by short inverted repeats (black arrows).** The LTRs contain elements U3, R and U5 for transcription initiation and termination. The primer binding site (PBS) and polypurine tract (PPT) are priming sites for reverse transcription (solid black lines). The PBS also matches a limited set of tRNAs. The universal 5’TG end and the CA 3’ terminus adjacent to the PBS are shown as small black boxes. The internal domain consists of *gag* and *pol* regions. The *gag* region encodes capsid-like proteins (CP) and has a nucleic acid binding moiety (NA). The pol region encodes protease (PR), integrase (INT), reverse transcriptase (RT) and RNase-H.

Transposable elements (TE) are mobile DNA sequences which can change their positions in the genome. Since their discovery in maize by Barbara McClintock [115] it has become evident that they are the largest components of most eukaryotic genomes [116,117]. Before discussing mobile element based markers in detail, some general points regarding classification and genomic organization must be addressed. This is of considerable importance given that these techniques utilize the specific features of different TEs and differ in the properties and annealing sites of the primers used within the transposable region. Based on their characteristics TEs have been divided into Class I (retrotransposons), commonly called ‘copy-and-paste’ elements, and Class II (DNA transposons), or ‘cut-and-paste’ elements [118]. Class I elements propagate via RNA intermediates and create an additional new copy in the genome, while Class II elements do not need an RNA intermediate and simply excise from the donor site of the genome and move to the novel position at the acceptor site. Since the discovery of many eukaryotic TEs such as miniature inverted repeat transposable elements (MITEs) this classification has been challenged, as it is hard to place the new elements in the existing system [119]. Wicker et al. [120] revised the scheme by maintaining the standard two-class system (as opposed to using enzymological features), but introduced hierarchical rankings which have become widely adopted for classifying TEs. In particular, Class I elements, retrotransposons, provide an excellent basis for the development of marker systems, since they share specific features relevant to primer design and genomic abundance due to their ‘copy-and-paste’ propagation. Most TE-based markers utilize Class I retrotransposons. In plants, LTR retrotransposons are widely distributed in the genome [121] and represent a family of eukaryotic TEs where the element is surrounded by long terminal repeats (LTRs). LTRs do not code for any protein but instead contain the promoters and terminators for transcription. These regions provide the basis for primer binding sites in many techniques. An LTR retrotransposon is shown in Figure 4.

#### Inter-retrotransposon amplified polymorphism (IRAP)

**Figure 5:**
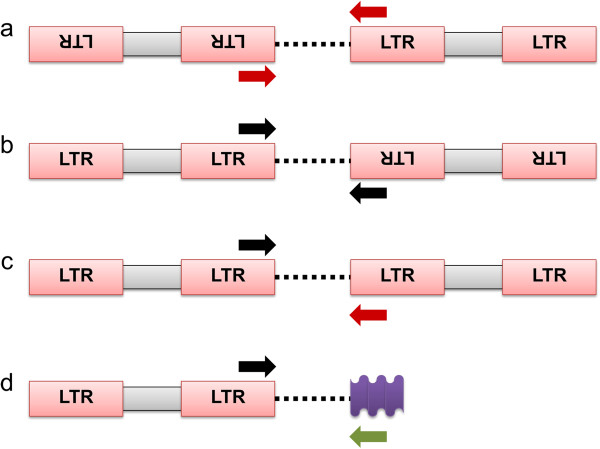
**An outline of inter-retrotransposon amplified polymorphism (IRAP).** Retrotransposons are in **a)** head-to-head, **b)** tail-to-tail or **c)** head-to-tail orientation. In the case of a) and b) only one primer is needed for a successful amplification while for the orientation illustrated in c) primer pairs are needed to generate banding patterns. **d)** The retrotransposon-microsatellite amplified polymorphism (REMAP) technique, where amplification takes place between a LTR retrotransposon and an adjacent microsatellite region. Red boxes indicate LTR motifs, internal domains are represented by grey boxes, and dashed lines show intervening genomic DNA. Color arrows designate primers, while the purple wavy box indicates a microsatellite region.

IRAP and REMAP are mobile element based marker systems described by Kalendar et al. [63] for generating DNA fingerprints. They both target a group of retrotransposons that contain direct long terminal repeats (LTRs) varying in size from 100–5,000 bp [122]. IRAP primers anneal to these regions and amplify DNA segments between two LTR sequences. Either one or two primers specifically designed for LTRs can be used in the same PCR, but the results will be determined by the orientation of these regions. The targeted Class I elements use the ‘copy-and-paste’ method of transposition which can take place in either orientation (5’ to 3’ or 3’ to 5’). Besides genomic abundance, this leads to differently oriented gene (copy) clusters found in head-to-head, tail-to-tail or head-to-tail orientation (Figure 5). For head-to-head and tail-to-tail arrangements, only a single primer is necessary to generate IRAP products (Additional file 4). For head-to-tail orientation, both 5’ and 3’ LTR primers are needed to amplify the intervening genomic DNA [63].

#### Retrotransposon-microsatellite amplified polymorphism (REMAP)

The other technique, REMAP, exploits polymorphisms among regions amplified between an anchored simple sequence repeat (SSR) and an LTR sequence (Figure 5). To achieve this, one specifically designed LTR primer is mixed with another arbitrarily chosen primer containing a simple repeat [e.g., (CA)_n_, (GA)_n_] plus an additional and randomly chosen anchoring nucleotide at the 5’ or 3’ end [e.g., C(CA)_n_, (GA)_n_G]. This technique can be regarded as a modified or extended version of the inter-simple sequences repeat (ISSR) technique, since one of the primers in a REMAP reaction is an anchored ISSR primer combined with an IRAP primer. IRAP and REMAP have been used individually and in combination to study genetic diversity in several plant genera, e.g., [123,124], because they produce reliable and reproducible banding profiles (Additional file 5).

#### Inter-SINE amplified polymorphism (ISAP)

This technique, developed by Seibt et al., [64] is based on retrotransposons that lack LTR motifs. It was specifically designed for potato. A recent study using bioinformatics tools identified Solanaceae-specific short interspersed element (SINE) families and subfamilies [125], with approximately 6500 copies of such elements being found. ISAP markers are based on the amplification of genomic sequences between adjacent SINE elements. Primers anneal to different positions within the SINE elements and are either outwardly or inwardly oriented. Specific primer design is achieved by consensus comparison of different Solanaceae SINE elements. As these elements are widespread in solanaceous plants they are readily transferable within species and genera. However, they have not yet been tested. Seibt et al. [64] describe their technique as reproducible and useful for potato variety genotyping. However, the distribution of the SINE families, the positions of the designed primers and conservation of the priming sites as well as homology of the SINE elements strongly influence the obtained information. This marker system may prove to be highly specific (Additional file 6), and while it may not become very popular in plant genetics generally, it represents a good attempt to utilize available genomic resources and databases. However, the design of ISAP primers requires extensive prior genomic information about SINE elements.

#### Inter-primer binding site (iPBS) amplification

**Figure 6:**
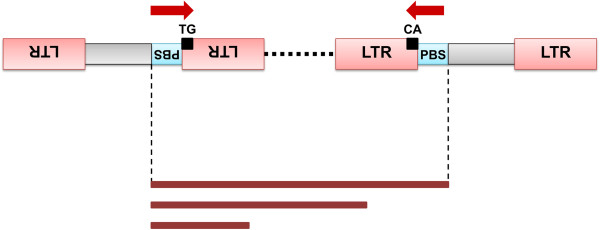
**Schematic representation of iPBS.** For successful amplification LTR retrotransposons must be in a head-to-head orientation. Primers are designed to anneal to the PBS regions (blue box), in the internal core (grey box), and have flanking regions in CA and TG in the LTR motif (red box). Different amplicons (brown bars) are generated containing the LTRs and the PBS regions plus the intervening genomic segment of variable length.

One of the limiting factors for utilizing retrotransposons as molecular markers is that LTR sequences must be known. If there is no *a priori* information, LTRs must be cloned and sequenced. The inter-primer binding site (iPBS) technique developed by Kalendar et al. [65] overcomes this problem by utilizing the PBS sites of retrotransposons that are shared by LTR transposons (Figure 4 and 6), with 18 nucleotides complementary to a limited set of tRNAs [126]. Primers of variable length (12–18 bp) are designed to anneal to these regions. For iPBS, the retrotransposons must have opposite directionality and be near enough to each other to amplify the intergenic regions. Since amplicons include the LTR motif the technique is also a very effective method for retrotransposon isolation and genome scanning. This could be very useful where additional fingerprinting markers (e.g., IRAP, REMAP) are needed or even where the diversity of TEs is the object of the study. Kalendar et al. [65] successfully tested their technique in studies of many plant species and also on many animal samples (Additional file 7). The method seems to be universal and transferable across many organisms in which retrotransposons have PBS elements [127,128].

#### Retrotransposon-based sequence-specific amplification polymorphism (SSAP)

**Figure 7:**
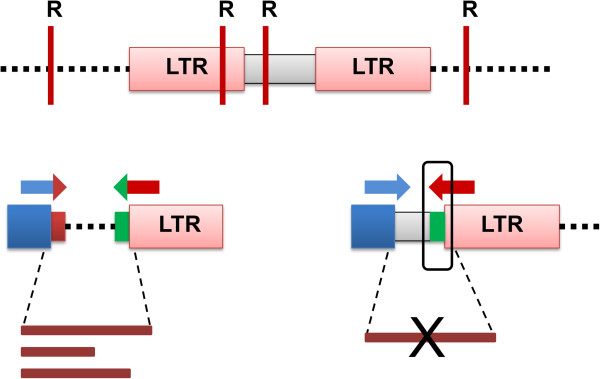
**An outline of SSAP.** DNA is digested with one frequently cutting and/or rarely cutting restriction enzyme (horizontal red lines marked ‘R’). Adapters (blue boxes) are ligated to restricted ends and then a pre-selective amplification is carried out (not illustrated). Selective PCR amplification, shown below, is carried out with LTR (red arrow) and adapter specific (blue arrow) primers. Both primers contain selective nucleotides (colored heads of the arrows) to ensure specific amplification and reduce the number of generated bands to a manageable level. Transposon amplification can only be carried out from the construct shown on the left as the primer at the 3’end contains a selective nucleotide that is absent from the one shown on the right. Generated PCR products of variable length are indicated by brown bars.

This technique, developed by Waugh et al., [66] is highly similar to amplified fragment length polymorphism (AFLP; [16]). It converts retrotransposon insertion sites into banding patterns using primers annealing to the junctions between the transposon and the host genome [129]. While for a typical AFLP procedure no *a priori* sequence information is required, careful planning and prior transposon sequence knowledge is strongly recommended for SSAP. Genomic DNA in SSAP is digested with an infrequently cutting restriction enzyme paired with a frequently cutting one (usually *Mse*I and *Pst*I, or any other restriction enzyme). After digestion, short double-stranded adapters (or adaptors) with known sequences are ligated to the restricted DNA fragments (Figure 7). This is followed by a pre-selective PCR amplification with adapter–homologous primers. The pre-amplification step is performed to reduce genome complexity and to ensure higher reproducibility. The next step is selective amplification with a retrotransposon specific primer, paired with either a rare or a frequent site adaptor primer. Primers usually anneal to retrotransposon LTR regions or to internal parts of the element. The sizes of the fragments are determined by the distance between the transposon insertion site and the adjacent restriction cut site, with differences in insertion sites between genomes easily visible as different banding patterns [130]. For SSAP amplifications, *Ty1-copia* or *Ty3-gypsy* retrotransposons are commonly used.

## Utility of mobile elements as molecular markers

Retrotransposons replicate by successive transcription, reverse transcription and insertion of the new cDNA copies back into the genome, very much like retroviruses. The structure and replication strategy of retrotransposons give them several advantages as markers, [63] as listed below.

### Abundance and copy number

Retrotransposons represent highly heterogeneous populations of elements in the genome and are widely dispersed in chromosomes, showing insertional polymorphism both within and among plant taxa [122]. It has been shown that LTR retrotransposons make up as much as 25% [116] of the maize genome (Meyers et al. 2001). Most plant genomes appear to contain LTR-retroelements in abundance [122]. However, it seems that their distribution and abundance is connected with genomic complexity, as plant species with smaller genomes tend to have a much smaller proportion of retrotransposons (e.g., < 5% in *Arabidopsis*[131]). In this regard, marker systems amplifying from fewer targets would result in less complex banding patterns. This phenomenon seems to be analogous to that observed in the case of AADs where there is an increase in genomic complexity, e.g., with polyploid formation, which is fairly common in plants. Therefore, the ability to detect polymorphism and the distribution of markers in the genome strongly depends on the chosen retroelement. As before, such issues are carefully investigated during primer design and genome coverage is not supposed to cause problems. The study of Manninen et al. [132] with IRAP and REMAP markers resulted in dense coverage of a 30 cM segment in barley chromosome 6H. This proved to be extremely useful in the identification of resistance loci against net blotch (*Pyrenophora teres* Drechs. f. *teres* Smedeg.). The same features are shared by SSAP, as markers generated and mapped in cashew (*Anacardium occidentale* L.) and in an interspecific cross of *Lactuca serriola* DH_M21(SER) (P1) × *L. sativa* cv Dynamite showed high levels of distribution [133,134]. Other techniques such as iPBS and ISNAP were developed for fingerprinting studies, but just as with IRAP and REMAP, application in linkage mapping may also be possible (Table 2).

### New genomic insertions, co-dominance and homoplasy

New insertions of mobile elements lead to polymorphism which can be detected and used to temporally order insertion events in a lineage [122]. Many types of mobile elements are widely distributed in the euchromatin domains of chromosomes, making it possible to generate markers linked to a given phenotype [135]. Moreover, mobile element based markers can be co-dominant. However, despite the fact that they are extremely useful for population genetics, all mobile element based markers have the same drawback: difficulty of data interpretation and uncertainty about the true nature of the polymorphism. Specifically, the question may arise as to whether differences in banding patterns are due to the absence or presence of retrotransposons, or are caused by some other mechanism, e.g., indels or restriction site loss. Fortunately, advances in analytical methods and a number of successful studies indicate that these drawbacks can be overcome. Different studies show that they generate both dominant and co-dominant markers and that the proportions of these seem to be variable [129,132,136]. Retrotransposon derived PCR products are amplified from a genome with a particular configuration of element insertions, but the PCR pattern resulting from a specific alternative allelic state, where a particular insertion is missing, is not *a priori* predictable [137]. The conservation of certain LTR regions facilitates the easy cloning and characterization of unique and co-dominant bands, which is a major advantage over AADs.

An important trait of mobile element markers is that homoplasy seems to be very rare [138]. Character states are clearly derived from a common ancestor and they are almost invariably identical by descent, but not identical by state [138]. Their ancestral state is known and stable, which means that the ancestral state at any amplified locus is the absence of the element, and once the element is present it will almost invariably remain there indefinitely [139]. It seems that most cases of homoplasy or mistakenly inferred homology arise from poor gel resolution or laboratory errors. These can manifest as poorly separated, unscorable bands, which are not identical in origin and represent different loci, or co-migrate among different samples. If the guidelines provided by Kalendar and Schulman [140] (applicable to most slab gel methods) are followed, these errors can be easily avoided.

## Resistance-gene based markers (RGMs)

Resistance-gene markers are a unique group within gene-targeted markers because they utilize specific features of genes involved in plant defense mechanisms [141,142]. Before discussing the details of these markers, it is necessary to briefly describe some common features of plant disease resistance. Plants have evolved active and passive defense mechanisms to protect themselves against pathogens. Active mechanisms comprise adaptive and innate types of immune responses. Adaptive immunity is based on the RNAi-type of response and functions mainly against viruses. Innate immunity is more general and enables the plant to defend itself against a large variety of pathogens by means of pathogen and pattern resistance receptors (PPRs) and resistance proteins (R proteins) [143,144]. PPRs recognize microbe or pathogen associated molecular patterns that are conserved among pathogens belonging to a particular class [145]. R proteins, in turn, recognize unique avirulence (Avr) factors that are not conserved among pathogens. R protein induced-signaling leads to production of reactive oxygen species and induction of a specific type of programmed cell death, termed the hypersensitive response, that destroys the affected cells [146]. The latest research indicates that cell death does not actually restrict the spread of the pathogen; instead its movement is blocked in the surrounding surviving tissue by an unknown mechanism [147]. R-protein mediated innate immunity is also termed gene-to-gene resistance, as each R gene responds to a specific pathogenic Avr gene [146]. Consequently, it is expected that a large number of R genes per plant genome are able to confer resistance against a large spectrum of pathogens. Also, R genes are under diversifying selection to keep pace with the rapid evolution of pathogens. Although different R genes respond to very different pathogens, they share several conserved regions (domains). Based on these domains, R proteins can be divided into four subclasses. The majority of R proteins contain a central nucleotide binding site (NBS) that acts as a molecular switch to control the activation status of the protein, and a C-terminal, leucine-rich repeat domain (LRR) which is required for Avr factor recognition. Thus, R protein division is based on variation in the N-terminal domain [148]. NBS-LRR type R proteins with N-terminals are homologous with *Drosophila* Toll and human Interleukin receptors and collectively they are all classified as TIR-NB-LRR proteins. Non-TIR NBS-LRR proteins are referred to as CC-NBS-LRR proteins, because some non-TIR proteins contain a coiled coil (CC) domain in their N terminus [149]. In addition, there are two classes of R proteins that contain an extracellular LRR in their N terminus. One of these classes, termed receptor like kinases (RLKs), contains a cytoplasmic protein kinase domain [150]. Receptor like proteins (RLPs) in turn lack this cytoplasmic protein kinase domain. As R genes from different plant species share conserved domains, they can be used to screen plant genomes for R genes and putative R genes (e.g., resistance gene analogs, RGAs), and to create molecular markers. This section focuses on the methods employed for R gene screening using PCR-based methods.

### Resistance-gene analog polymorphism (RGAP)

RGAP employs uncut genomic DNA as a PCR template and degenerate primers for conserved regions of R genes to screen for R genes and RGAs [67]. Over a decade ago in studies of crop species, it was shown that agarose gel electrophoresis is insufficient to detect the majority of PCR fragment length polymorphisms in highly heterogeneous PCR product pools [151]. However, denaturing polyacrylamide gel electrophoresis (PAGE) yields up to a 130-fold increase in fragment length polymorphism separation capability. PAGE has been subsequently used for PCR band separation in the majority of plant profiling studies. Based on the results of Leister et al., [67] accurate PCR markers linked to R-genes can be quickly obtained using R-like gene specific primers. RGAP has been shown to be feasible in several areas of research. It has been used in a number studies to create molecular markers for R genes that confer resistance to pathogens, e.g., [152,153]. It has also proven to be useful in biodiversity studies for characterizing R gene domains (namely, NBS and LRR domains) and for analyzing genetic variability (see Additional file 8).

### Nucleotide-binding site (NBS) profiling

**Figure 8:**
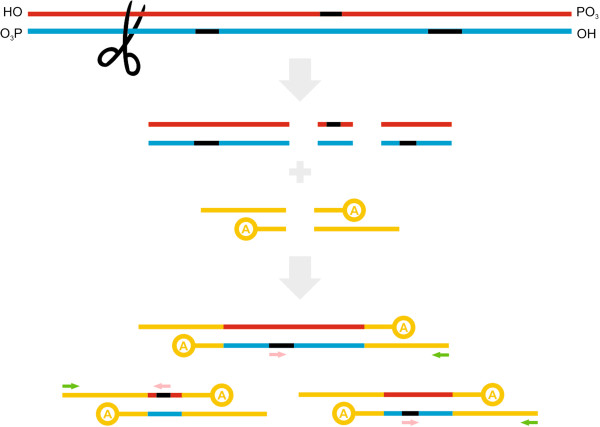
**Diagrammatic representation of NBS profiling.** Genomic DNA is cut with a single restriction enzyme that creates blunt-ended fragments. Asymmetric adapters containing short arms that are blocked with an amino group (denoted by A) to prevent extension by DNA polymerase are ligated to the ends of the fragments. Primers specific for NBS and the adapter long arm are used to amplify fragments containing the NBS sequence. Additional preceding asymmetric PCR is also often performed with the adapter primer and a limited amount of the NBS-specific primer, or alternatively only with the latter to increase the efficiency of NBS-specific amplification (see text for details). Black segment – NBS; pink arrow – NBS-specific primer; green arrow – primer specific for adapter long arm.

Linden et al. [68] described an advanced NBS profiling approach based on conserved NBS amplification and demonstrated its feasibility in a variety of plants (potato, tomato *Solanum lycopersicum* L., barley, and lettuce) in screening for R genes and RGAs. In this approach, genomic DNA is restricted with a single restriction enzyme that creates blunt-ended fragments (in contrast to SSAP and AFLP where a rarely cutting and frequently cutting enzyme combination is used). Asymmetric adapters containing short and long arms are ligated to the ends of restriction fragments (Figure 8). The 3’ end of the short arm is blocked with an amino group to prevent extension by DNA polymerase and decrease amplification of adapter-adapter fragments. Fragment amplification is performed in two steps. Firstly, a linear PCR is performed with only the NBS-specific degenerate primer. It is advisable to keep primer degeneracies low, and to avoid degeneracy within the last two positions in the 3’ end of the NBS primer. The linear PCR product is used as a template in a second exponential PCR with NBS-specific and adapter-specific primers. The adapter primer sequence is identical to the adapter long arm, ensuring the selective amplification of only those fragments, previously amplified during the NBS-specific linear PCR. NBS profiling has been used for a number of purposes (Additional file 3). It was initially used for mapping R genes and RGAs alone or in combination with two other molecular marker technologies (SSAP and AFLP). NBS profiling has proven its superiority over AAD marker techniques in the quantification of genetic variation. The potential of NBS profiling has also been exploited in phylogenetic analyses (see Additional file 9). Interestingly, NBS profiling yielded comparable results to AFLPs in this study [154].

## Advantages of resistance-gene based markers

### Easy transferability and high specificity

Resistance-gene based techniques have the advantage of being able to create molecular markers linked to potentially functional genes [155]. They generate specific fragments; nearly 90% of all bands are amplified from R genes or RGA related regions. Primers with lower RGA amplification rates seem to generate fragments outside of the highly conserved NBS domain [154]. Within the NBS domain, sequence conservation is high, whereas outside of it conservation between different RGAs is much lower [68]. However, sequencing the amplified bands suggests that the rate of non-specific bands generated by RGM methods is underestimated [154]. In some cases non-specific bands appear to be loci that are not represented in GenBank, and therefore it is hard to assign them to any RGA cluster. This is true especially for underutilized plant species. Furthermore, targeted R genes represent a very important class of plant genes, with important roles in creating profitable breeding programs and studying plant biodiversity and evolution. Sequence information is not required prior to analysis, as R gene profiling employs locus-specific degenerate primers targeting highly conserved R gene domains. Since primers for RGMs are conserved they can be easily transferred to virtually any plant taxon, facilitating cross-species amplifications. Furthermore, resistance-gene derived fragments can be further analyzed and converted to cleaved amplified polymorphic sequence (CAPS) and sequence-characterized amplified region (SCAR) markers [152,153,156]. The study of Valkonen et al. [157] showed that these markers can be reliably used in marker assisted selection (MAS), since they are tightly linked to resistance-gene like sequences. This can be a major advantage over other gene-targeting markers such as retroelement-based markers, where prior sequence information is required to position the primers. This advantage has increased the popularity of AAD markers in a diverse range of plant groups. As alternatives to other techniques, RGMs can be used where no prior genomic knowledge or even no sequence information is available. Such markers can be used to assess genetic diversity among resistance loci, or to characterize germplasm collections based on these traits (Table 3). The effective characterization of the gene pools of wild relatives of crop species using RGM methods could highly beneficial. It could facilitate the management of genetic resources, as plant breeding programs are mostly concerned with finding and introgressing traits - mostly resistance genes - found in wild relatives. Moreover, resistance gene clusters often undergo recombination and insertion/deletion events leading to the generation of new specific variants of resistance to pathogens [158,159], which can be easily detected with resistance-gene based markers.

**Table 3:** Comparison of various aspects of resistance-gene based markers and RNA-based markers

	**Resistance-gene based markers (RGMs)**			**RNA-based markers (RBMs)**		
	**RGAP**	**NBS-profiling**	**iSNAP**	**cDNA-AFLP**	**cDNA-RFLP**	**EST-SSR**
Abundance	High	High	High	High	High	Medium
Reproducibility	Medium	High	High	High	High	High
Polymorphism	Medium	High	High	High	Medium	Medium
Prior sequence information	No	No	Yes	No	No	Yes
Visualization	Silver stained PAGE	Silver stained PAGE	Silver stained PAGE	Silver stained PAGE	Silver stained PAGE	Silver stained PAGE
Specificity	High	High	High	Medium	High	High
Size of bands	200-1,500 bp	70-600 bp	100-1,500 bp	100-1,000 bp	100-3,500 bp	100-400 bp
Homoplasy	Low	Low	Not reported	Medium	Low	Medium
Reaction artifacts						
i. Uniparental bands	No	No	Not reported	Rare	No	No
ii. Heteroduplexes	Not reported	No	May occur	No	No	Yes
iii. Nested priming	May occur	No	May occur	No	No	No
iv. Other	Generated bands may evolve under selection		No	No	No	No

### Low level of homoplasy and utility in systematics

An important requirement for phylogenetic studies is that inferences should be based on homologous characters that share common ancestry. Strictly homologous molecular characters or orthologous sequences are often assumed to map to the same genomic location, while paralogs map to different positions. However, orthologous sequences could also map to different positions due to extensive genomic rearrangements [160]. Therefore, it is better to view homology as a relationship based on common origin between any entities without further distinction, [161] while orthology is descent from a single ancestral sequence with relationship viewed in terms of speciation (vertical descent). Paralogy, by contrast, can be viewed as relationship via duplication [162]. Many multi-locus methods fail to fulfill the requirement for homology as they produce non-homologous bands that are mistakenly inferred to be homologs after phylogenetic analysis. In this case the scored bands are apparently similar but phylogenetically independent. In DNA fingerprinting apparent homology may arise from non-identical bands that co-migrate simply by chance or because they share similar sequences, but these can be either orthologs, pseudogenes, transposable elements or even repetitive elements with unknown functions [163]. False scoring of just slightly different size fragments in two separate profiles can also lead to false homology [164]. In this respect, the problem of correct homology assessment may not be restricted to phylogenetics but may be a factor in all genome scanning studies. In the case of resistance-gene based banding patterns it can be difficult to define characters as either orthologous or paralogous. Genetically linked gene families have higher probabilities for recombination than single genes. Genetic recombinations between alleles of R genes of the same cluster can re-assort the genetic variation created by mutation to create new alleles [159]. The importance of this in R genes is illustrated by the fact that most novel alleles are associated with recombination events [158]. In the reciprocal arms race of host parasite evolution a number of factors affect the degree to which the members of an R gene cluster recombine with each other to create new variants. Although the resistance-gene families are regarded as stable complexes, unequal recombination occurs, albeit only at low frequencies. In the case of some unexplained scenarios, unequal recombinations can be implicated as sources of homoplasy. However, homoplasy becomes a greater problem when distantly related species are involved and is less likely to be a problem for studies of very closely related species with a similar genomic organization [39,40]. The targeting of more conserved regions of resistance-genes makes RGMs more appropriate for many applications, since the chance of homoplasy is reduced. It has been shown in the Zingiberaceae that NBS markers score over SSRs since they are highly conserved [165]. This may be due to several factors such as constraints on allele size range, high mutation rates, size homoplasy and low levels of conservation of SSRs among Zingiberaceae, which hampered the use of microsatellites in this study. Other results have demonstrated that systematic relationships inferred from NBS-profiling data may not be essentially different to those derived from AFLP [166], or RAPD data [167]. In these studies, the patterns generated by NBS-profiling complimented the results obtained from the other markers systems. Similar comparisons for RGAP have not yet been made. This indicates that resistance-gene based markers can be at least as useful as AADs or SSRs for phylogeny reconstruction, and they may even perform better when more diverse material is used due to a reduction in the levels of homoplasy. As paralogy depends on the mutation rate of the RGAs it may be possible that bands are non-homologous. If co-migrating non-homologous bands do exist in resistance-gene based fingerprints their frequency must be low due to the specificity of amplification as discussed in the previous section. However, the drawbacks of using degenerate primers may yet remain, as these specific primers may nonetheless be biased towards known R genes. However, it has been shown that although R gene profiling yields genes that are already known, plenty of new RGAs are also targeted [68].

### The evolution of resistance-genes is under selection

Functional sequences are assumed to be under selection. Plants do not have circulatory system based immunity as is seen in animals. Therefore, they are very dependent on individual cellular defense mechanisms, which are often based on single R-genes with specific structures. These genes are likely to be under selection, which might influence the outcome of any phylogenetic analysis. Results indicate that different regions of these genes evolve with different rates according to a birth-and-death process [168]. Some regions are hypervariable and incorporate many non-synonymous and synonymous mutations, while other parts evolve at a steadier rate. Resistance-gene based fingerprints are preferentially generated from plant resistance genes; therefore they better shape the evolution of these genes within a species, or among certain taxa. In the case of tuber-bearing *Solanum* species, poor resolution was obtained at the basal nodes of the reconstructed phylogenetic trees based on NBS-profiling [154]. The authors explained this by extensive hybridization among species that evolved within a relatively short period of time, coupled with rapid radiation with no clear sequential branching. This observation may indicate that R gene evolution and species evolution could be linked, and banding patterns may reflect true phylogenies. R genes with different selection mechanisms may occur in a specific profile at a relatively low frequency, but these few bands will not significantly affect the overall phylogeny. These single resistance genes in some cases could be crucial for the survival of a species at a particular moment of speciation. On an evolutionary time scale this would equate to a short period as plant pathogens spread rather fast, requiring that the resistance genes necessary for survival should also spread rapidly. According to Wang et al., [154] the specific effect of selective pressure on R-genes will therefore only be detectable on a very short evolutionary time scale, and would be diluted when many markers are analyzed phylogenetically.

## RNA-based markers (RBMs)

Biological responses of plant cells to certain stress factors are important phenomena, as these processes depend on the regulation of gene expression. Many methods have been developed in an attempt to gain an insight into these processes, and this has led to the generation of PCR-based markers. Fingerprinting markers are based on the specific amplification of a subset of fragments, which can be derived from RNA as well as DNA. The techniques summarized here are based on transcribed regions of the genome that are most likely functional. Recently, Gupta and Rustgi [1] reviewed molecular markers derived from the transcribed/expressed regions of genomes. These are treated here also if they utilize cDNA or ESTs. The methods described here may utilize the RNA pool directly, or after further processing, using cDNA or ESTs coupled with bioinformatic tools to generate random or specifically designed primers.

### Inter small RNA polymorphism (iSNAP)

**Figure 9:**
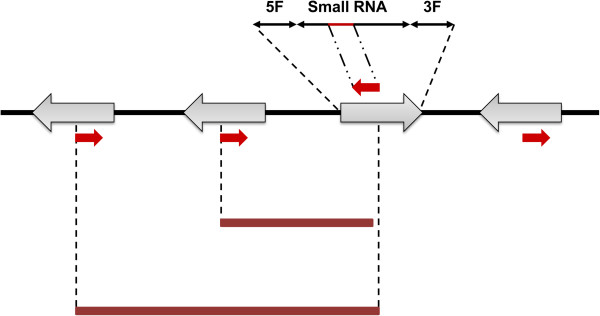
**Outline of iSNAP.** Differently oriented small RNAs (grey arrows) are present in the genome. Primer design can be carried out either from core small RNAs (red bar) or 5’ and 3’ flanking regions. These primers can be used to generate fingerprints either solely (as presented on the figure) or in combination. Successful amplifications depend on the orientation of small RNAs in the genome. PCR products are depicted as brown bars.

Endogenous non-coding small RNAs consisting of 20–24 nucleotides are ubiquitous in eukaryotic genomes, where they play important regulatory roles, [69] and they provide an excellent source for molecular marker development. The flanking sequences of small RNAs are conserved, allowing the design of primers for use in PCR reactions and fingerprinting (Figure 9). The technique developed by Gui et al., termed iSNAP, [69] exploits this feature. The basic principle is to use primer pairs of flanking small RNAs to initiate a PCR reaction and detect length polymorphisms that are due to indels present in the small RNA pool [169]. According to the authors the technique is reproducible, representing a high-throughput, non-coding, sequence-based marker system. It can be used for genome mapping and for genotyping.

### cDNA-AFLP

This method was developed by Bachem et al. [70] and consists of four major steps for generating fingerprints. Firstly, RNA is extracted from plant tissues, which is then used for cDNA synthesis. Further steps are similar to the protocols for AFLP and include restriction digestion with one or two restriction enzymes, with the cDNA used as a primary template. The digestion is followed by the ligation of adapters and anchors. After this a preamplification is carried out with primers corresponding to the anchors. In the final step a selective amplification is implemented, with extended primers having one or even more selective nucleotides. The resulting fingerprints are visualized by silver-staining of polyacrylamide, or else fluorescently labeled primers can be used to detect peaks. This technique is efficient for the identification of common and rare transcripts and for studying genome-wide gene expression [170]. It can also be used to identify differences in the expression of different genes under various stress conditions [171]. Since the initial description of the basic techniques, many modifications have been published that have increased the efficiency of the method [170,172,173]. Using cDNA-AFLP a genome wide transcriptome map has been constructed for *Arabidopsis*, [174] and it has also successfully been used to detect gene expression alterations in *Triticum aestivum*[175] and to develop polymorphic transcript-derived fragments (TDFs) in *Manihot esculenta* Crantz [176].

### cDNA-RFLP

The study of Bryan et al. [71] showed that cDNA clones can also be directly used as probes for RFLP analysis. These markers can be converted to specific PCR markers, and these genome-specific amplicons used in gene tagging or diagnostics. Subsequent studies have modified the basic technique by altering the probes, or the way that the probes are generated for the analysis. Probes can be designed in such a way that permits applications across species or even across genera [177] within a particular plant family [178]. Another possibility is to use probes from PCR products amplifying cDNA products of specific genes [179]. Alternatively, the cDNA clones can be used directly without any screening for RFLP analysis. This method has been used effectively in several plant species such as sunflower (*Helianthus annuus* L.; [180] and wheat [181].

### EST-SSR

Sequencing of cDNA produces a large amount of information, now available in public databases. Expressed sequence tags (ESTs) are short transcribed sequences that are usually read in a single direction and provide a good basis for gene expression analyses and detecting genetic diversity. Once converted to cDNA the expressed genes can be sequenced in two directions, producing 5’ and 3’ ESTs. The latter fall more often within untranslated regions (UTRs), while 5’ ESTs are associated with protein coding. Many available bioinformatics tools, e.g., [72,182], allow these databases to be easily searched to develop EST-based molecular markers. The recent increase in the availability of expressed sequence tag (EST) data has facilitated the development of microsatellite or simple sequence repeat (SSR) markers in a number of plant species groups [183]. Technically, EST-SSRs do not differ from common genomic (gSSR) microsatellites in their amplification or detection. The major difference is in primer development and the locations of the primers, as EST-SSRs are generated from the transcribed region of the genome. They are harvested directly from sequence data using *in silico* techniques. Data mining can be carried out in many alternative databases specifically designed for particular plant groups, e.g. Triticeae, [184] or more commonly in NCBI-EST [185]. There are many software tools specifically designed for database mining, e.g., SSRFinder [186], BuildSSR [187], and TRF [188]. Further examples can be found in the review by Varshney et al. [189]. Expressed sequence tag derived genic SSRs are most likely to be found within functional sequences, and thus provide abundant information compared to genomic SSR markers. Their most important feature is easier transferability among distantly related species compared with gSSRs. Such markers can be used for the same purposes as gSSRs and have proved to be useful in the analysis of alpine lady-fern (*Athyrium distentofolium* Tausch ex Opiz; [190], rice, [191] and the genus *Medicago* L. [192].

## Advantages of RNA-based markers

Plant genetic programs aiming to characterize the transcribed region of the genome yield a large amount of ESTs, genes and cDNA clones directly accessible from different databases developed for these purposes. In most cases the major aim of these studies is not the generation of new marker sets, or the development of primers based on novel sequence information, but rather analysis of (for example) plant stress responses. However, marker development can benefit from such approaches as new primers from the expressed region of the genome can be developed with bioinformatics tools and algorithms. In this regard cDNA or EST derived markers are no more than byproducts of large sequencing projects that can be sorted by bio-data mining. Such processes can be carried out relatively easily and without significant costs if free software is used for data processing. Once ESTs are generated and used for different purposes new primers can be developed cheaply. The same applies to iSNAP markers, as these were also developed based on the results of large scale next generation sequencing of small RNAs. The greatest advantage of RBMs is that they are derived from the expressed region of the genome. The generated fragments can easily be associated with phenotypic traits, this being extremely important for genetic mapping studies. On the other hand, in studies aiming to explore genetic variation in natural populations these markers should be used with caution, because they may be under selection. RNA-based markers are also expected to be transferable between related species and genera as the primers are designed from conserved coding regions of the genome. As iSNAP is recent technique, information is still sparse. Easy transferability of the EST-derived markers has been demonstrated in several studies [193-195]. The consensus finding of these studies is that EST-derived markers can be applied without any redundancy in related plant genera, even in cases where detailed sequence or EST information is lacking. However, in cross-species applications the recurring problem of orthology assessment can arise. Studies suggest that primers designed for a given species will most probably amplify the same fragment in related genera [189]. The amplification success rate seems to vary among different plant groups. In the genus *Medicago*, 96% of primers designed for *M. truncatula* Gaertn. generated fragments in other species of the genus, suggesting unproblematic interspecific transferability [192]. Results are more variable at the intergeneric level, as only 59% of tall fescue (*Festuca arundinacea* Schreb.) primers amplified in rice, while better results (71%) were obtained for the same primers in wheat [196]. Success of transfer may be related to genomic complexity, taxonomic distance, and the function/evolution of the gene from which the EST primers are derived. Due to their robustness, the development of EST-derived markers is especially popular in crop breeding programs, especially in cereals, where large genomic libraries exist and ESTs are more frequently used compared with other crop species [189]. Genetic diversity research programs exploring the wild relatives of economically important crop species have a particular opportunity to benefit from these developments. Unfortunately, the same cannot be said for other plant genera that lack economic importance despite having ecological or evolutionary significance. A summary of various aspects of RBMs can be found in Table 3.

## Targeted fingerprinting markers (TFMs)

Taking advantage of the increasing knowledge of genomic elements, a novel family of markers has been developed, here termed targeted fingerprinting markers (TFMs). These are by definition multi-locus markers, generated in a semi-random and targeted manner from various regions of the genome, and presumably corresponding to polymorphic sites of any gene or gene related region irrespective of their function. This means that marker systems grouped here are (gene)-targeted markers which do not necessarily yield fingerprints involved in phenotypic trait variation. TFM markers tend to combine advantageous features of several basic techniques, while also incorporating methodological modifications to increase sensitivity and resolution in order to detect genetic discontinuity and distinctiveness. They incorporate modifications of the primers and benefit from *a priori* genomic information available for the organism. Anchoring elements (e.g., gene promoters or start codons) are added to various parts of the primers to ensure directed amplification of gene-related regions or sites flanking the targeted region. Fingerprints are generated in a semi-random manner, because due to the incorporation of common features of the plant genome, banding patterns are produced from anonymous but targeted sites. This enables whole genome distribution and better reproducibility than can be achieved with specific primer design or even with modified PCR protocols. Exploiting common genomic features makes TFM techniques easily transferable between many organisms and provides alternatives to previous AAD markers. They differ from each other with respect to important features such as genomic abundance, level of polymorphism detected, locus specify, reproducibility, technical requirements, and cost. The major TFM techniques will be summarized here according to their requirements and the modifications that characterize them.

### Direct amplification of length polymorphisms (DALP)

**Figure 10:**
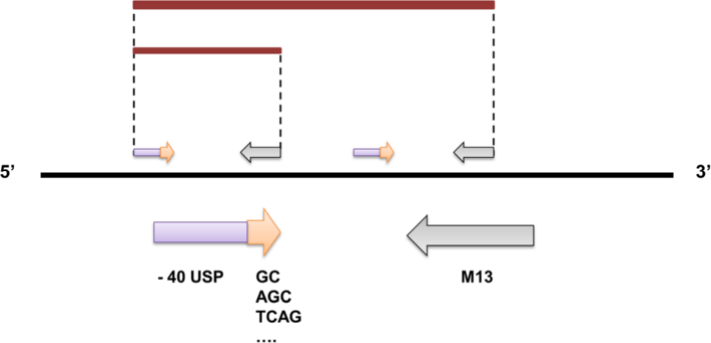
**Outline of the DALP technique.** For DALP fingerprinting, a universal M13 sequencing primer (grey arrow) is used as a reverse primer. The forward primer includes a – 40 USP core region (purple box) and variable selective nucleotides at the 3’ (orange head of the arrow).

This technique, developed by Desmarais et al., [73] resembles AAD but detects a larger number of polymorphisms and simplifies the procedure for recovering the resulting banding patterns. It also has the advantages of high-resolution fingerprinting in that it offers the possibility of directly sequencing each new marker locus [197]. It was designed to obtain nucleotide sequence information for DNA fragments from any genome with no *a priori* sequence data (Figure 10). For PCR amplifications, the universal sequencing primer ‘M13 – 40 USP’ is incorporated in the oligonucleotide set as a core. Selectivity is ensured by adding further bases to the 3’ end of the primers, which are termed ‘selective primers’. The reverse primer is also a common ‘M13’ which is standardly used in primer paired reactions. Primer sets with any desired length can be designed by varying the composition of 3’ bases in the selective primer. This technique is an explicit extension of RAPD with longer primers (19–21 bp). The main advantage of the method is that banding patterns (Additional file 10) are obtained with a minimum number of primers by simple combinations and by changing only one primer between different experiments. Studies utilizing DALPs report that results can be reliably and rapidly obtained for a wide variety of purposes, including investigation of population diversity [105,198], genetic mapping [197] and defining new monolocus co-dominant markers [199].

### Promoter anchored amplified polymorphism (PAAP)

Promoter regions facilitate gene transcription and are located close to a particular gene, [200] therefore they can be used to specifically profile the genome of the analyzed organism. Promoter elements determine the point of transcription initiation and alter the rate and specificity of transcription [201]. The gene specific architecture of promoter sequences shows high diversity, consisting of many short motifs that serve as recognition sites for proteins involved in transcription initiation [202,203]. This feature of promoters makes them suitable for tagging with degenerate primers to generate length polymorphisms, easily detectable by electrophoresis. Pang et al. [74] designed several short oligonucleotide primers containing the degenerate sequence of cotton (*Gossypium* L.) promoter regions. They named the technique promoter anchored amplified polymorphism (based on random amplified polymorphic DNA, PAAP-RAPD), as the primers can either be used alone or in combination with common sets of RAPD primers. It is relatively difficult to characterize promoter regions in different organisms, but numerous databases (e.g., PlantProm [201]) exist which can help in the design of further primers for various purposes. The authors imply that the technique might be useful for developing molecular markers to search for polymorphism associated phenotypic traits amplified from the regulatory regions of plant genomes.

### Sequence-related amplified polymorphism (SRAP)

**Figure 11:**
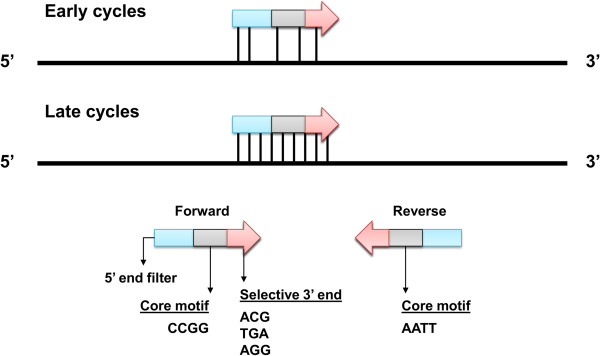
**Outline of SRAP.** In the SRAP reaction each primer contains a random filter sequence at the 5’end (blue box) and three variable selective nucleotides at the 3’ end. The core motif consists of CCGG in the forward primer and AATT in the reverse primer, both targeting gene related regions. Early cycles of PCR are carried out at a lower temperature (35°C) allowing mismatches to be incorporated in the amplicons. Late cycles at a higher annealing temperature (50°C) generate products from this pre-amplified pool.

A large number of polymorphisms can be revealed using primers targeting short recognition sites in the plant genome, since almost any primer can initiate PCR amplification. Region amplified polymorphic (RAP) techniques also use arbitrary primers, but differ significantly from the widely used RAPD technique [14]. Based on the modifications incorporated in the primers, three main techniques have been developed. The first of these was sequence-related amplified polymorphism (SRAP), developed by Li and Quiros [75]. The primers used in this technique are longer (17–21 nt) than the 10 nt ones used in RAPD. The forward and reverse primers contain GC and AT-rich sequences near the 5’ and 3’ ends, respectively. This is based on the rationale that protein coding regions tend to contain GC-rich codons, while 3’ UTRs frequently consist of AT-stretches [204]. The same authors noted that approximately one-third of the *Arabidopsis* genome found in chromosomes 2 and 4 represents exon regions containing the ‘CCGG’ motif. With the inclusion of this motif in the core of the forward primer, exon regions containing this element are preferentially amplified. Because exons are generally conserved and might fail to produce sufficient polymorphism, the reverse primer in SRAP is designed to contain a second core with the aforementioned ‘AATT’ motif, which is frequently found in promoters, introns and spacers. Since these regions are more variable between different individuals, the intrinsic dissimilarity incorporated in the primer sets makes it feasible to generate polymorphic bands based on introns and exons [75]. The arbitrary primers also contain further modifications of 10 bases at the 5’ end called filter sequences, with no specific constitution. These are followed by the core sequences (CCGG for forward and AATT for reverse), while at the 3’ end three selective nucleotides are added. The PCR profile is also modified to ensure specificity and high stringency and consists of two parts, the early and late cycles (Figure 11). Primer-DNA template annealing depends on the matching level of both sequences determining the amplification efficiency. Using this characteristic of PCR, many mismatch amplicons are generated during initial early cycles at a lower annealing temperature (35°C). The low initial annealing temperature ensures the binding of both primers to sites with partial matches in the target DNA, creating a population of amplicons that contains the priming sites. During the late cycles at a higher annealing temperature (50°C), the initially generated amplicons serve as templates rather than the genomic DNA, ensuring high reliability, efficiency and reproducibility due to perfect base pairing of primers with the template. Because mismatches are allowed in the early cycles, the 5’ ends of the PCR primers are usually ‘forced’ into the PCR products. This is similar to *in vitro* mutagenesis using PCR primers [205]. For a successful amplification the 3’ sequences of the primers are crucial, and should match perfectly during the PCR cycles [206]. Therefore the 3’ limits the amplifications leading to polymorphic alleles with perfect 3’ matches and rejecting alleles with mutations in these regions from the population of amplicons generated during the early cycles. New polymorphic sites can be easily generated (Additional file 11) by varying the selective nucleotides at the 3’ ends. SRAP has rapidly gained in popularity based on the following advantages: i) a large number of polymorphic fragments are amplified in each reaction, ii) there is no *a priori* need for information about sequences, iii) primers can be applied to any species, iv) it is cost effective and easy to perform, v) reproducibility is high, and vi) PCR products can be directly sequenced using the original primers without cloning. The method has now been widely used in plant genetics (see Additional file 3).

### Targeted region amplified polymorphism (TRAP)

The second technique, called Targeted Region Amplified Polymorphism (TRAP) and developed by Hu and Vick [76], is similar to SRAP but is based on *a priori* sequence information. The PCR conditions are the same as described for SRAP, with the priming and amplification procedure having the same rationale. The PCR reaction consists of a fixed and an arbitrary SRAP primer incorporating the aforementioned modifications, i.e., selective nucleotides, filter sequences and AT- or GC-motifs. The fixed primer is designed from available partial sequences of candidate genes, such as expressed sequence tags (ESTs). The generation of fixed primers limits the use of this technique to species where ESTs are known, or requires the generation of new sequence information for primer development (Additional file 12). Despite this limitation it has been widely used for several purposes in different plant species, e.g., [207,208]. Based on the use of ESTs to design primers, this method could also be placed in the RNA-based markers group, although it shares many common features with SRAP.

### Conserved region amplification polymorphism (CoRAP)

CoRAP [77], is also based on the use of a fixed and an arbitrary primer. While TRAP resembles SRAP in using the same arbitrary primer, CoRAP is much more similar to TRAP since it also uses a fixed primer derived from directly targeted ESTs. The only difference is in the arbitrary primer, which contains a different core sequence motif (CACGC), commonly found in plant gene introns. This core sequence ensures the utilization of conserved intron sequences in plant genotyping while the fixed (conserved) primers target coding sequences, together generating highly reproducible and reliable fingerprints. The advantage of TRAP and CoRAP is that the fixed primers derived from ESTs will have specific binding sites on the exon of the target sequence, while the arbitrary primers will bind to most of the introns (CoRAP), or to other exon regions (TRAP), during the PCR amplifications. If the distribution of these gene elements allows successful PCR, banding patterns resulting from a specific fingerprint will be amplified. Indels in these regions will certainly generate different distributions of amplified products. The closer the genetic relationship between the two individuals, the more similar the corresponding band patterns of the amplified PCR products will be [77].

### Start codon targeted (SCoT) polymorphism

Molecular markers from the transcribed region of the genome have potential for various applications in plant genotyping as they reveal polymorphism that might be directly related to gene function. A novel marker system called Start Codon Targeted Polymorphism (SCoT) quickly gained popularity after being described by Collard and Mackill [78]. This method is based on the observation that the short conserved regions of plant genes are surrounded by the ATG translation start codon [209]. The technique uses single primers designed to anneal to the flanking regions of the ATG initiation codon on both DNA strands. The generated amplicons (Additional file 13) are possibly distributed within gene regions that contain genes on both plus and minus DNA strands. The utility of primer pairs in SCoTs was advocated by Gorji et al. [210]. SCoT markers are usually reproducible, while primer length and annealing temperature are not the sole factors determining reproducibility [104,210]. They are dominant markers, however, while a number of co-dominant markers are also generated during amplification, and thus could be used for genetic diversity analysis. SCoTs can be used either in isolation or in combination with other techniques to assess genetic diversity and to obtain reliable information about population processes and structure across different plant families [211].

## Characteristics of TFM markers

### Reproducibility and increase of complexity with polyploidy

In some cases reproducibility can be a problem with techniques that detect large amounts of polymorphism or more complex banding patterns (Table 4). However, with careful PCR optimization reproducibility need not be a severe problem. It is well known that polyploidization can promote rapid essential rearrangements in the genome such as genome restructuring, intergenomic recombination, or even a rapid loss of DNA [212]. As TFMs are generated semi-randomly and/or yield functional gene region related banding patterns spanning the entire genome, it seems clear that these techniques are influenced by genomic rearrangements. The application of such multi-locus markers in the same way as AADs can produce incorrect genetic distances depending on the degree of genomic rearrangement. Based on the results of Poczai and Hyvönen [213], genetic distances between hypothetical parental diploids and their derived allopolyploids estimated by PCR-based multi-locus banding patterns will increase. Unfortunately, studies including a detailed investigation of the effects of polyploidy on banding patterns are very rare for TFMs. For very complex banding patterns bands should be separated on polyacrylamide gels rather than agarose, as suggested in the descriptions of the TFM methods.

**Table 4:** Comparison of various aspects of targeted fingerprinting markers

	**Targeted fingerprinting markers (TFMs)**					
	**DALP**	**PAAP**	**SRAP**	**TRAP**	**CoRAP**	**SCoT**
Abundance	High	High	High	Medium	Medium	High
Reproducibility	Medium	Low	High	High	Medium	Medium
Polymorphism	High	Medium	High	High	Medium	Medium
Prior sequence information	No	No	No	Yes	Yes	No
Visualization	Silver stained PAGE	Agarose gel electrophoresis	Silver stained PAGE or agarose gel electrophoresis	Silver stained PAGE	Silver stained PAGE	Agarose gel electrophoresis
Specificity	Low	Low	High	High	Medium	Low
Size of bands	200-1,000 bp	200-1,200 bp	50-1,500 bp	50-900 bp	50-900 bp	200-1,500 bp
Homoplasy	Medium	High	Medium	Medium	Not reported	High
Reaction artifacts						
i. Uniparental bands	May occur	May occur	May occur	No	No	May occur
ii. Heteroduplexes	Yes	Yes	May occur	May occur	Yes	May occur
iii. Nested priming	Yes	Yes	Yes	Yes	Yes	Yes
iv. Other	Complexity of banding patterns may increase with polyploidy					
	Limited independence of bands	Limited independence of bands	Limited independence of bands	No	Not reported	Limited independence of bands
	Not reported	No	Overlooked co-dominancy	Overlooked co-dominancy	Overlooked co-dominancy	Overlooked co-dominancy

### Independence of TFM markers

Detailed information on the independence of bands generated by TFMs is practically non-existent, unlike with AADs. The independence of scored markers is limited by linkage, as they should be derived from separate loci if they are not to be regarded as dependent (here meaning that the locus is counted more than once). Dependence is important for some studies, since loci scored in such a way could be easily overlooked. For genetic mapping the behavior of the markers is an important feature, for example AADs tend to cluster in the pericentromeric regions and although they are randomly generated, tend to form clusters when the constructed genetic map becomes denser [214,215]. The behavior of some TFMs in mapping studies is well documented, while for some other markers this information is still lacking. For example, SRAP markers showed even distribution on the linkage map constructed for *Brassica oleracea* (not differing from the results obtained with AFLP) in the study of Li and Quiros [75]. SRAP markers showed more consistent distribution in other studies, which may indicate that they are better markers than AFLPs for map construction [216,217]. This must be due to the fact that AFLP is affected by DNA methylation, resulting in pseudo-polymorphism and uneven marker distribution in some species [218]. Another interesting feature of SRAPs is that they can form groups in linkage maps where AFLP, SSR and RFLP markers frequently form dense clusters. Lin et al. [219] showed that SSRs and RAPDs were generally distributed between SRAPs, with an even distribution within and among linkage groups. For genetic diversity assessment of germplasm collections, SRAP markers are also considered to be superior to AADs as they seem to be more congruent with morphological variation and evolutionary history [220]. TRAP markers show similar features to SRAPs, but in polyploid genomes TRAPs are unequally distributed among some homologous groups [221]. Moreover, in sunflower, Hu [222] was able to define linkage groups in telomeric regions. DALP markers also appear to be a good complement to AFLP in linkage mapping, having similar features to those described above for SRAP and TRAP. The characteristics of SCoT, PAAP and CoRAP markers in this type of study remain unknown.

### Simultaneous occurrence of dominant and co-dominant bands

A marker can become dependent based on overlooked co-dominancy or nested priming. The latter is easier to detect, while undetected co-dominancy may lead to an overestimate of the number of polymorphic loci and an underestimate of allelic diversity [27]. Any co-dominant bands discovered should be coded in a multi-allelic system, and analyzed in a different manner from binary dominant data. It has been reported that SRAP yields dominant and co-dominant markers together in the same reaction. The frequency of co-dominant bands seems to vary among taxa. Li and Quiros [75] found that 20% of all scored bands were co-dominant. They emphasize this finding as being an important advantage of this technique over AADs. The same phenomenon was also reported for DALP and TRAP [199,223,224]. However, examples where no co-dominant bands have been found are also known [225]. The ability of TFMs to generate co-dominant bands should not be overestimated, as they never exceed 20% – the frequency is thus moderate rather than high. Other marker systems, including CoRAP, PAAP and SCoT, are also based on the same rationale, in the sense that they are also (gene)-targeted markers incorporating modifications in the primers. In this respect they should in theory detect both dominant and co-dominant markers, as has been reported for other techniques of this marker group, but such experimental evidence is still lacking for CoRAP and PAAP. In the case of SCoT some data are available, and indicate mixed presence of both markers types in generated banding patterns [213,226]. Gorji et al. [210] also noted that the shared absences of some SCoT bands represented inversions of shared presences for all individuals in a mapping population of tetraploid potato, possibly belonging to different alleles of the same locus. This could be a good starting point for providing sequence level evidence. Additional *in silico* analysis could also be carried out with test organisms where the sequence of the entire genome is known and only a few chromosomes need to be covered with markers, for example *Arabidopsis*. Such a study has already been performed with AFLP, and concluded that centromeric enrichment of *Sac*I/*Mse*I AFLP markers is due to higher levels of nucleotide substitution in non-coding than in coding regions [227].

## Concluding remarks

Although the use of some recently developed marker techniques in plant science is not yet as extensive as that of well established methods such as AADs, the number of studies utilizing these advanced methods is increasing. This may be attributed to the fact that these marker systems have the potential to provide new sources of information. Some recently developed techniques can be regarded as under-utilized tools for researchers, and as yet none have become as popular as RAPD or AFLP, despite the fact that they have been shown to be as or more effective than these traditional techniques. Major efforts have been made to develop new and more efficient markers for plants of agricultural importance (e.g. potato, rice, maize), but much less research has focused on developing markers for underutilized crops. Some marker techniques are still not available in other scientific fields, such as molecular ecology and phylogenetics, where the organisms of interest lack economic importance and there is no prior sequence or genomic information available for primer design. A major disadvantage of some recently developed methods is the need for preliminary genomic information, which in some cases requires additional and time-consuming laboratory work. As the costs of DNA sequencing fall with the advent of high-throughput methods the costs of developing gene-targeted markers will be reduced. The increasing number of studies based on recently developed marker systems suggests that such techniques could be useful for many different purposes. In addition, these methods seem to be more specific than AADs, which are mostly based on unknown and sometimes extensive genomic rearrangements. It can be expected that most of the methods discussed here could provide more structured datasets which could be used alone or in combination with sequence level characters in certain fields of plant biology where they have not yet been utilized.

## Abbreviations

AAD: Arbitrarily amplified DNA marker;AFLP: Amplified fragment length polymorphism;CAPS: Cleaved amplified polymorphic sequence;CDDP: Conserved DNA-derived polymorphism;CDM: Conserved DNA and gene family based marker;CISP: Conserved-intron scanning primers;CoRAP: Conserved region amplification polymorphism;cTBP: Combinatorial tubulin based polymorphism;Cyt P450: Cytochrome P450 mono-oxygenases;DALP: Direct amplification of length polymorphism;EPIC: Exon-primed intron-crossing PCR;EST: Expressed sequence tag;FM: Functional marker;GTM: Gene-targeted marker;h-TBP: Horse tubulin based polymorphism;iPBS: Inter-primer binding site amplification;IRAP: Inter-retrotransposon amplified polymorphism;ISAP: Inter-SINE amplified polymorphism;ISJ: Intron splice junction;iSNAP: Inter small RNA polymorphism;ISSR: Inter-sample sequence repeat;IT: Intron-targeting;ITP: Intron-targeting polymorphism;LTR: Long terminal repeat;MAS: Marker assisted selection;MITE: Miniature inverted repeat transposable element;NBS: Nucleotide binding site;PAAP: Promoter anchored amplified polymorphism;PBA: Cytochrome P450 based analogues;PBS: Primer binding site;PIP: Potential intron polymorphism;PLUG: PCR-based landmark unique gene;RAPD: Random amplified polymorphic DNA;RBM: RNA-based markers;REMAP: Retrotransposon-microsatellite amplified polymorphism;RGA: Resistance gene analogs;RGAP: Resistance-gene analog polymorphism;RGM: Resistance-gene based markers;SCAR: Sequence-characterized amplified region;SCoT: Start codon targeted polymorphism;SINE: Short interspersed element;SRAP: Sequence-related amplified polymorphism;SSAP: Retrotransposon-based sequence-specific amplification polymorphism;SSCP: Single-strand conformation polymorphism;SSR: Simple sequence repeat;TBP: Tubulin based polymorphism;TDF: Transcript-derived fragment;TE: Transposable elements;TFM: Targeted fingerprinting markers;TRAP: Targeted region amplified polymorphism

## Competing interests

The authors declare no competing interests.

## Authors’ contributions

**PP** conceived the study and drafted the early version of the manuscript. **IV**, **ML** and **ACs** researched chosen marker groups for the extended draft. **JH**, **JV, and NB** commented on the manuscript, revised the text and structure, and outlined it several times together with **PP**. All authors read and approved the final manuscript.

## Authors’ information

**PP**’s research focuses on phylogenetics and genomics, molecular ecology, especially Solanaceae. **IV** is a plant pathologist currently working with a hyperparasitic fungus infecting mistletoe. She studies the usability of this fungus in biological control and the diversity of the fungal populations. **ML**’s research interest is focused on gene technology and developmental biology. **ACs** is currently working on the transfer of effective alien genes into cultivated wheat from related species (barley, rye, *Aegilops* sp.) using classical genetic methods, molecular markers and cytogenetic analysis. **NB** studies phylogeny and macroevolution in mosses, focusing on Austral pleurocarpous groups, the Polytrichopsida and latterly the evolution of leaf structure. **JV** studies mechanisms of resistance to plant pathogens, especially viruses. **JH**’s research focuses on taxonomy of bryophytes, especially mosses, and the Polytrichopsida in particular. He has also participated in various cladistic analyses of diverse groups of embryophytes and fungi.

## Supplementary Material

Additional file 1: Figure S1 Anticipated results of TBP fingerprinting in different plant species. Primers and PCR conditions described in Breviario et al. [60]; bands were separated on 2% agarose gels. Plant species in each lane: 1. *Triticum aestivum* L., 2. *Zea mays* L., 3. *Hordeum vulgare* L., 4. *Glycine max* (L.) Merr., 5. *Avena sativa* L., 6. *Lolium italicum* A. Braun, 7. *Medicago sativa* L., 8. *Bromus hordeaceus* L., 9. *Poa pratensis* L., 10. *Arrhenatherum elatius* (L.) P.Beauv. ex J.Presl & C.Presl, 11. *Festuca arundinacea* Schreb., 12. *Holcus lanatus* L., 13. *Phalaris arundinacea* L., 14. *Dactylis glomerata* L., 15. *Poa trivialis* L.; Mm indicate the molecular marker size ladder (bp). Photo provided by Diego Breviario. Click here for file

Additional file 2: Figure S2 Intron-targeting fingerprint with *Ry*-*In4* primers in potato (*Solanum tuberosum* L.) population mapping. Bands separated on 1.5% agarose gel. Molecular marker size ladder is displayed on both sides of the lanes. Click here for file

Additional file 3: Table S1 Major application areas of gene-targeting and functional markers discussed in the study. Click here for file

Additional file 4: Figure S4 Utility of IRAP for a diversity analysis of a plant species. IRAP fingerprints of 30 genotypes of populations of *Hordeum spontaneum* K.Koch shown as negative images of ethidium bromide - stained agarose gels following electrophoresis. Results for BARE-1 LTR primer 1369 (5’– TGCCTCTAGGGCATATTTCCAACAC – 3’) are shown. A 100 bp DNA ladder is present on the left. Photo from Ruslan Kalendar and Alan Schulman. Click here for file

Additional file 5: Figure S5 Utility of REMAP for a diversity analysis of plant species. REMAP fingerprints of genotypes of populations of *Hordeum spontaneum*. Results are shown for BARE-1 LTR primer 1369 (5’–GGAATTCATAGCATGGATAATAAACGATTATC– 3’) and ISSR (5’– CACCACCACCACCACCACCACT – 3’). Photo from Ruslan Kalendar and Alan Schulman. Click here for file

Additional file 6: Figure S6 ISAP-Pattern of ten potato (*Solanum tuberosum*) varieties. Patterns generated with primers SolS-IIIa-F/SolS-IV-R and resolved on 2% agarose gel in 1×TAE buffer. 100 bp Plus Marker (M); varities Valisa (1), Venezia (2), Vienna (3), Vineta (4), Vitara (5), Vitesse (6), Wega (7), Zorba (8), Django (9), Europrima (10). Photo provided by Thomas Schmidt. Click here for file

Additional file 7: Figure S7 iPBS fingerprinting of apple (*Malus domestica* Borkh.) cultivars and their sports. Lanes are of the cultivars: 1, Atlas; 2, its sport Red Atlas; 3, Sävstaholm; 4, its red sport Bergius; 5, Syysjuovikas; 6, its sport Luotsi; 7, Melba; 8, its sport Melba Red Pate. Photo from Ruslan Kalendar and Alan Schulman. Click here for file

Additional file 8: Figure S8 RGAP patterns generated by the primer combination XLRRfor/XLRRrev. Samples were taken from different individuals of a Nicaraguan population of *Pinus oocarpa* Schiede ex Schltdl. Photo from Esther Ferrer. Click here for file

Additional file 9: Figure S9 An overview of NBS profiling NBS2/Rsa. To the left of the size marker are the lanes from tuber-bearing *Solanum* L. species, to the right lanes from different potato varieties. Photo by Miqia Wang, Gerard van der Linden and Ben Vosman (unpublished). Click here for file

Additional file 10: Figure S10 DALP fingerprints from different cultivated sunflower (*Helianthus annuus* L.) recombinant inbred lines. Fingerprints were generated with primer combinations DALP reverse (5’-TTTCACACAGGAAACAGCTATGAC-3’) and selective primer DALP-235 (5’-GTTTTCCCAGTCACGACCAC-3’). Photo kindly provided by Kamel Langar and André Bervillé. Click here for file

Additional file 11: Figure S11 SRAP fingerprints generated for *Brassica napus* L. genotypes. Products were amplified with fluorescently labeled primers analyzed with an ABI 3100 DNA analyzer. The virtual gel shown on the picture was produced with ‘Genographer’. Photo kindly provided by Genyi Li and Carlos Quiros. Click here for file

Additional file 12: Figure S12 TRAP profile of worldwide collected *Lactuca serriola* L. germplasm accessions. This primer set, F4RGC (fixed primer) + ODD15 (arbitrary primer), produced 35 polymorphic fragments with lengths varying between 0.1 kb and 0.9 kb. Such profiles can be useful for estimating genetic diversity and geographical relationships. Photo provided by Soon Jae Kwon. Click here for file

Additional file 13: Figure S13 SCoT profile generated from *Solanum* species. Bands generated with primer SCoT36 (5’-GCAACAATGGCTACCACC-3’) and separated on 1.5% agarose gel. Fingerprints are shown as a negative image of the ethidium-bromide stained gel. Click here for file
