# Supplementary figures and images for: Advances in plant gene-targeted and functional markers: a review
Source: Plant Methods. 2013 Feb 13;9:6. doi: 10.1186/1746-4811-9-6 (PMC3583794; doi:10.1186/1746-4811-9-6)

1 2 3 4 5 6 7 8 9 10 11 12 13 14 15 Mm

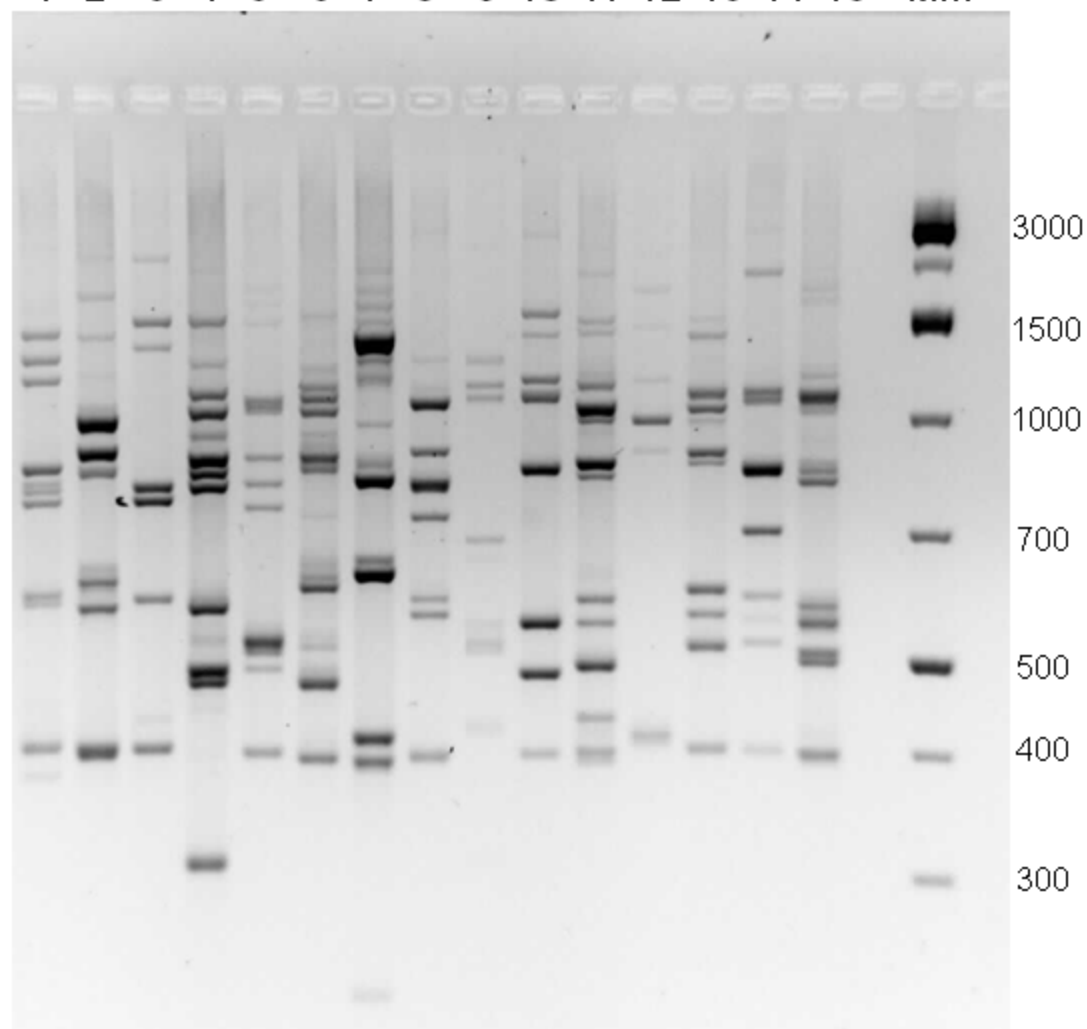

Supplement: Additional file 1: Figure S1 — Anticipated results of TBP fingerprinting in different plant species. Primers and PCR conditions described in Breviario et al. [60]; bands were separated on 2% agarose gels. Plant species in each lane: 1. Triticum aestivum L., 2. Zea mays L., 3. Hordeum vulgare L., 4. Glycine max (L.) Merr., 5. Avena sativa L., 6. Lolium italicum A. Braun, 7. Medicago sativa L., 8. Bromus hordeaceus L., 9. Poa pratensis L., 10. Arrhenatherum elatius (L.) P.Beauv. ex J.Presl & C.Presl, 11. Festuca arundinacea Schreb., 12. Holcus lanatus L., 13. Phalaris arundinacea L., 14. Dactylis glomerata L., 15. Poa trivialis L.; Mm indicate the molecular marker size ladder (bp). Photo provided by Diego Breviario. [file 1746-4811-9-6-S1.pdf]

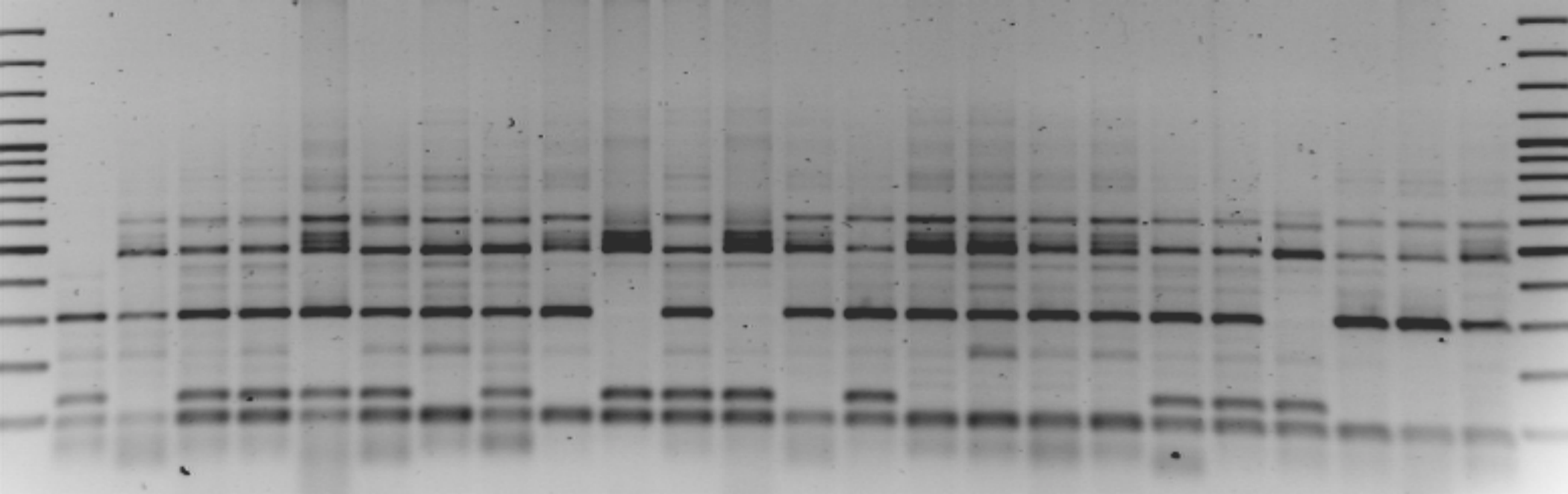

Supplement: Additional file 2: Figure S2 — Intron-targeting fingerprint with Ry-In4 primers in potato (Solanum tuberosum L.) population mapping. Bands separated on 1.5% agarose gel. Molecular marker size ladder is displayed on both sides of the lanes. [file 1746-4811-9-6-S2.pdf]

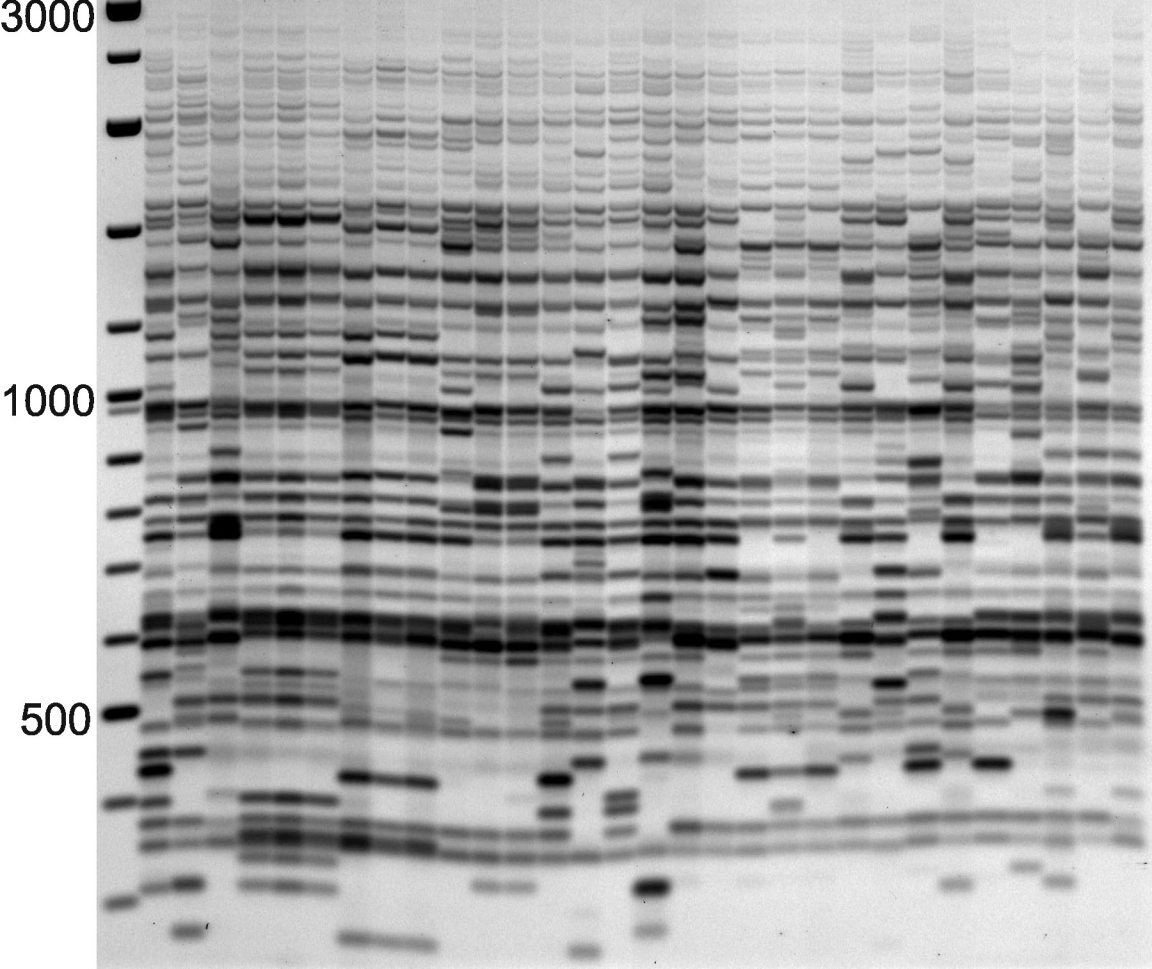

Supplement: Additional file 4: Figure S4 — Utility of IRAP for a diversity analysis of a plant species. IRAP fingerprints of 30 genotypes of populations of Hordeum spontaneum K.Koch shown as negative images of ethidium bromide - stained agarose gels following electrophoresis. Results for BARE-1 LTR primer 1369 (5’– TGCCTCTAGGGCATATTTCCAACAC – 3’) are shown. A 100 bp DNA ladder is present on the left. Photo from Ruslan Kalendar and Alan Schulman. [file 1746-4811-9-6-S4.pdf]

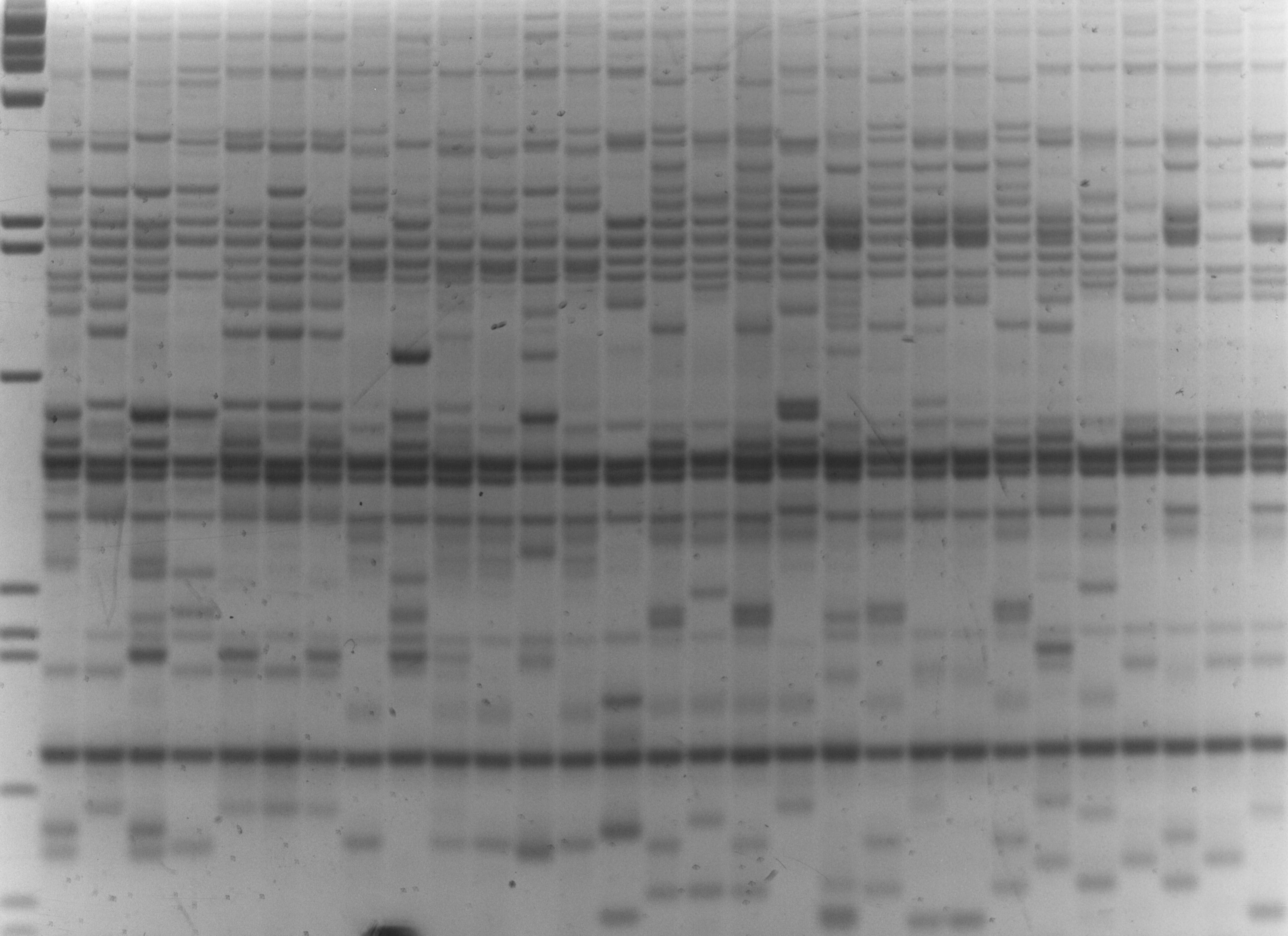

Supplement: Additional file 5: Figure S5 — Utility of REMAP for a diversity analysis of plant species. REMAP fingerprints of genotypes of populations of Hordeum spontaneum. Results are shown for BARE-1 LTR primer 1369 (5’–GGAATTCATAGCATGGATAATAAACGATTATC– 3’) and ISSR (5’– CACCACCACCACCACCACCACT – 3’). Photo from Ruslan Kalendar and Alan Schulman. [file 1746-4811-9-6-S5.pdf]

M 1 2 3 4 5 6 7 8 9 10 M

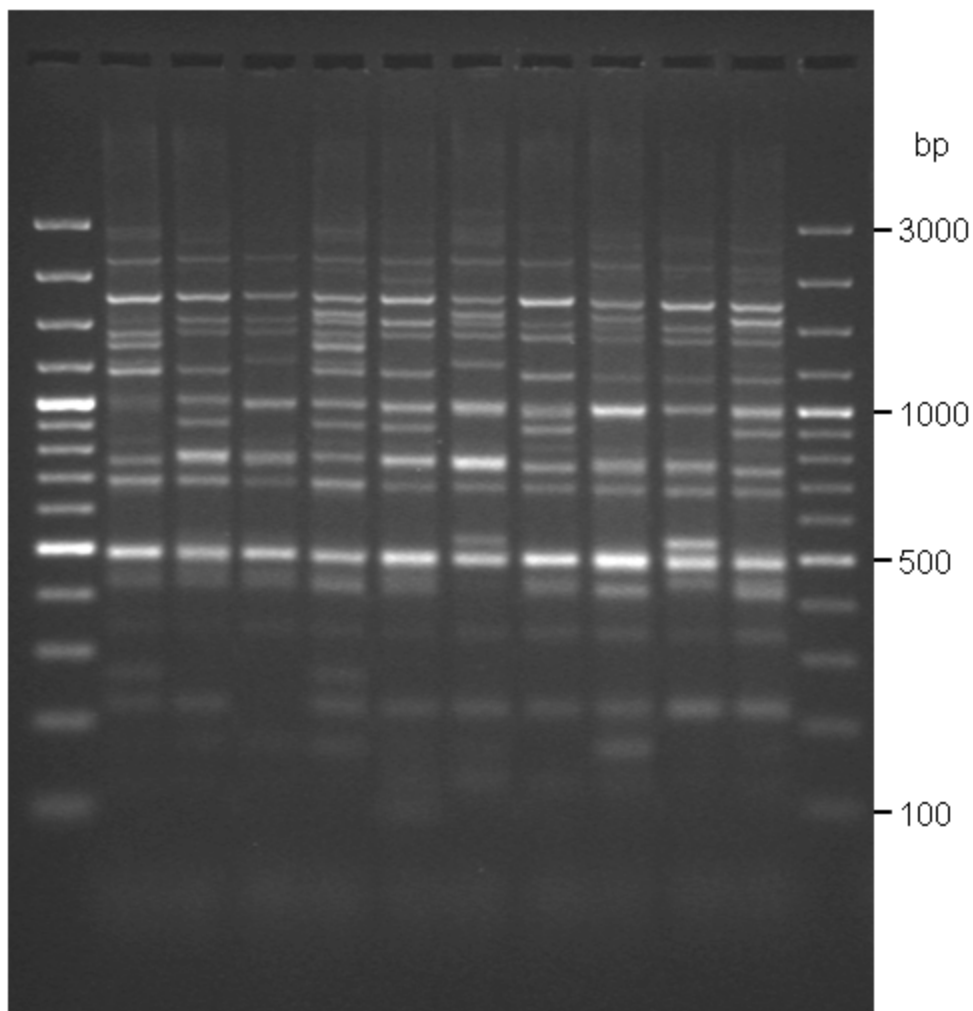

Supplement: Additional file 6: Figure S6 — ISAP-Pattern of ten potato (Solanum tuberosum) varieties. Patterns generated with primers SolS-IIIa-F/SolS-IV-R and resolved on 2% agarose gel in 1×TAE buffer. 100 bp Plus Marker (M); varities Valisa (1), Venezia (2), Vienna (3), Vineta (4), Vitara (5), Vitesse (6), Wega (7), Zorba (8), Django (9), Europrima (10). Photo provided by Thomas Schmidt. [file 1746-4811-9-6-S6.pdf]

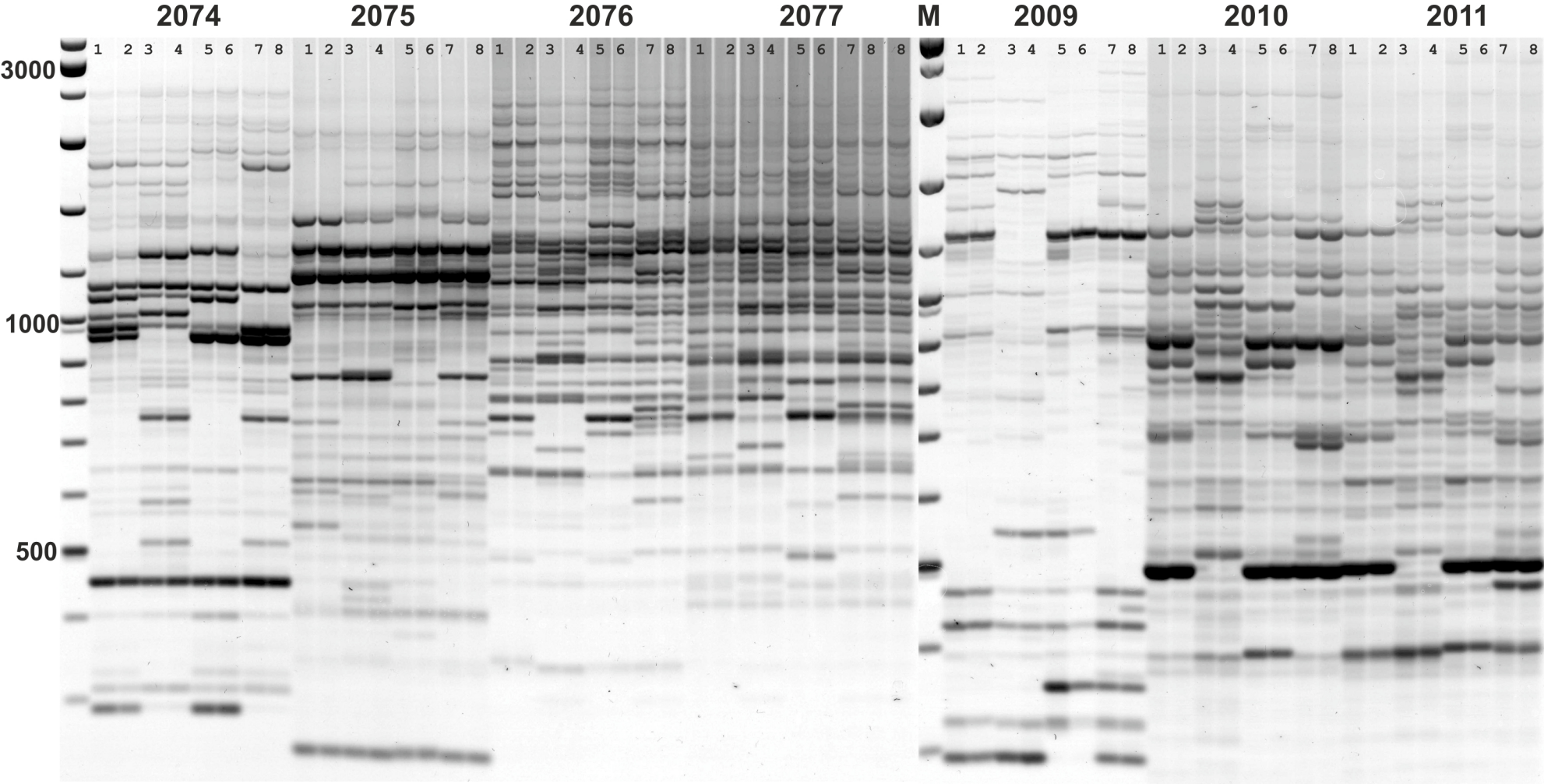

Supplement: Additional file 7: Figure S7 — iPBS fingerprinting of apple (Malus domestica Borkh.) cultivars and their sports. Lanes are of the cultivars: 1, Atlas; 2, its sport Red Atlas; 3, Sävstaholm; 4, its red sport Bergius; 5, Syysjuovikas; 6, its sport Luotsi; 7, Melba; 8, its sport Melba Red Pate. Photo from Ruslan Kalendar and Alan Schulman. [file 1746-4811-9-6-S7.pdf]

bp

1000

850

650

500

400

300

200

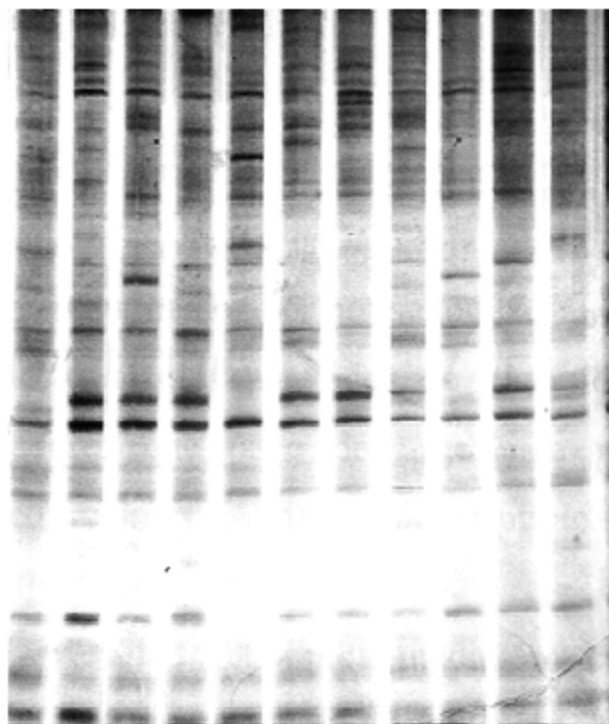

Supplement: Additional file 8: Figure S8 — RGAP patterns generated by the primer combination XLRRfor/XLRRrev. Samples were taken from different individuals of a Nicaraguan population of Pinus oocarpa Schiede ex Schltdl. Photo from Esther Ferrer. [file 1746-4811-9-6-S8.pdf]

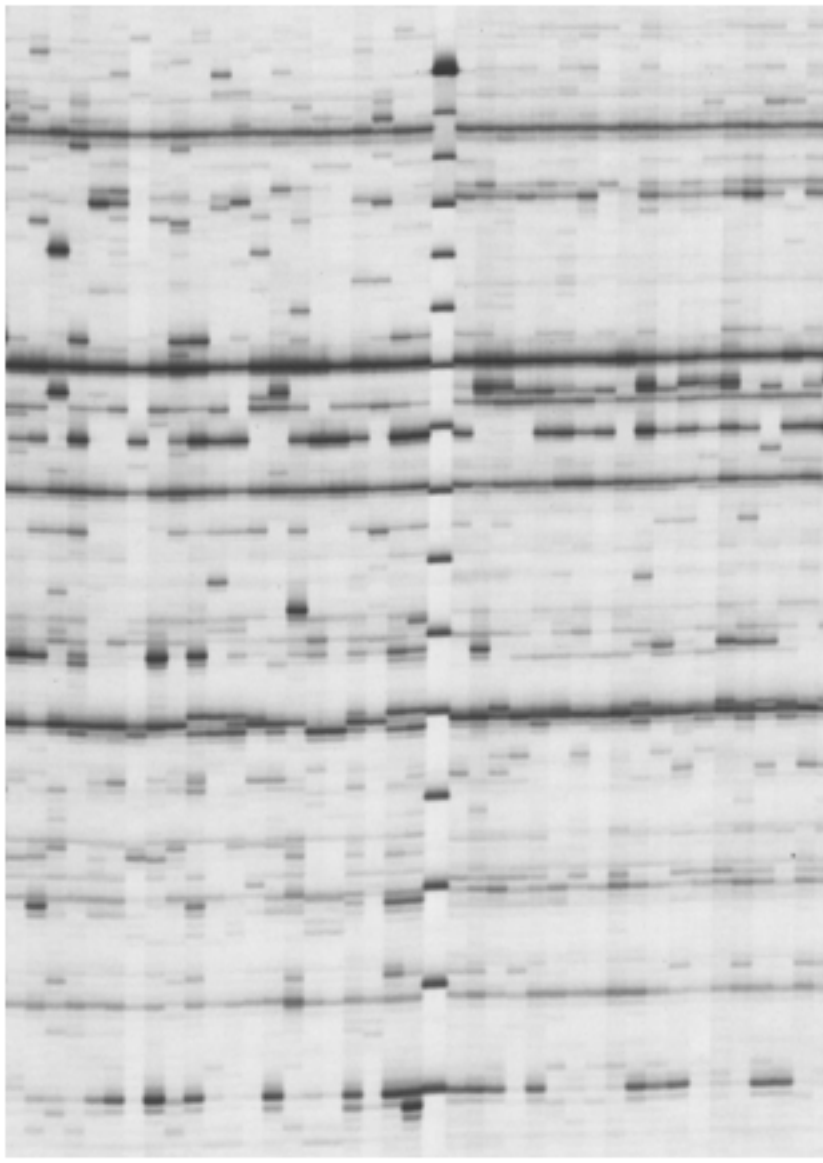

Supplement: Additional file 9: Figure S9 — An overview of NBS profiling NBS2/Rsa. To the left of the size marker are the lanes from tuber-bearing Solanum L. species, to the right lanes from different potato varieties. Photo by Miqia Wang, Gerard van der Linden and Ben Vosman (unpublished). [file 1746-4811-9-6-S9.pdf]

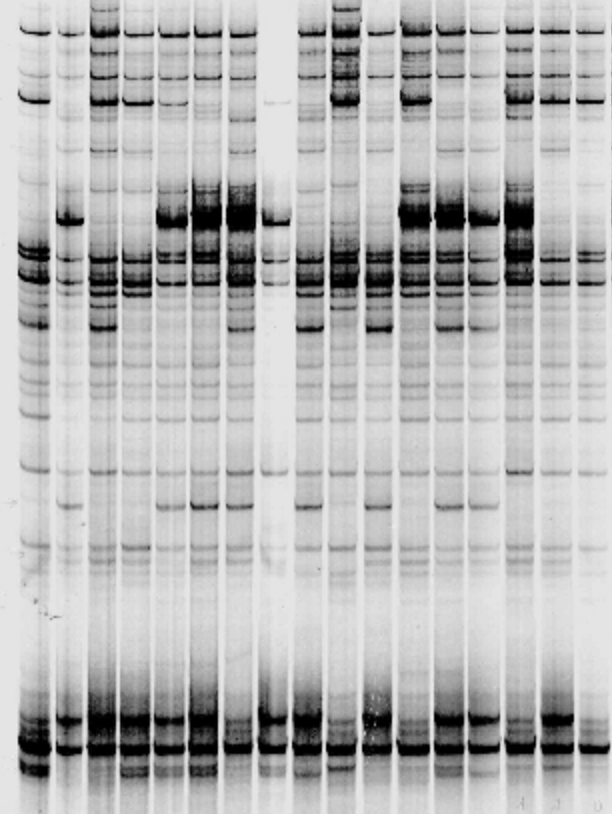

Supplement: Additional file 10: Figure S10 — DALP fingerprints from different cultivated sunflower (Helianthus annuus L.) recombinant inbred lines. Fingerprints were generated with primer combinations DALP reverse (5’-TTTCACACAGGAAACAGCTATGAC-3’) and selective primer DALP-235 (5’-GTTTTCCCAGTCACGACCAC-3’). Photo kindly provided by Kamel Langar and André Bervillé. [file 1746-4811-9-6-S10.pdf]

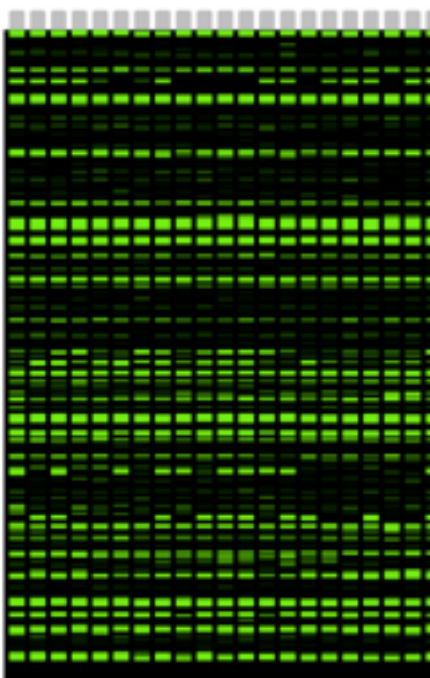

Supplement: Additional file 11: Figure S11 — SRAP fingerprints generated for Brassica napus L. genotypes. Products were amplified with fluorescently labeled primers analyzed with an ABI 3100 DNA analyzer. The virtual gel shown on the picture was produced with ‘Genographer’. Photo kindly provided by Genyi Li and Carlos Quiros. [file 1746-4811-9-6-S11.pdf]

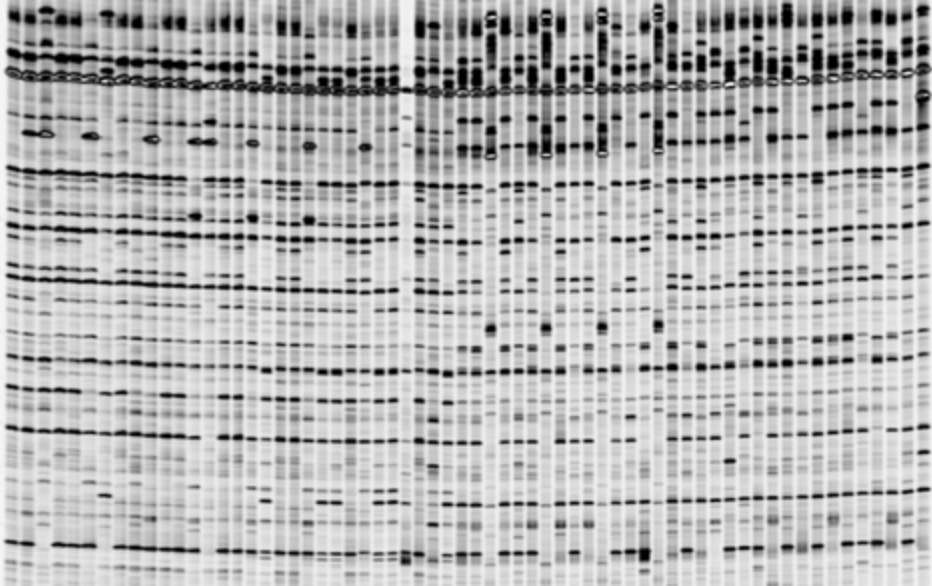

Supplement: Additional file 12: Figure S12 — TRAP profile of worldwide collected Lactuca serriola L. germplasm accessions. This primer set, F4RGC (fixed primer) + ODD15 (arbitrary primer), produced 35 polymorphic fragments with lengths varying between 0.1 kb and 0.9 kb. Such profiles can be useful for estimating genetic diversity and geographical relationships. Photo provided by Soon Jae Kwon. [file 1746-4811-9-6-S12.pdf]

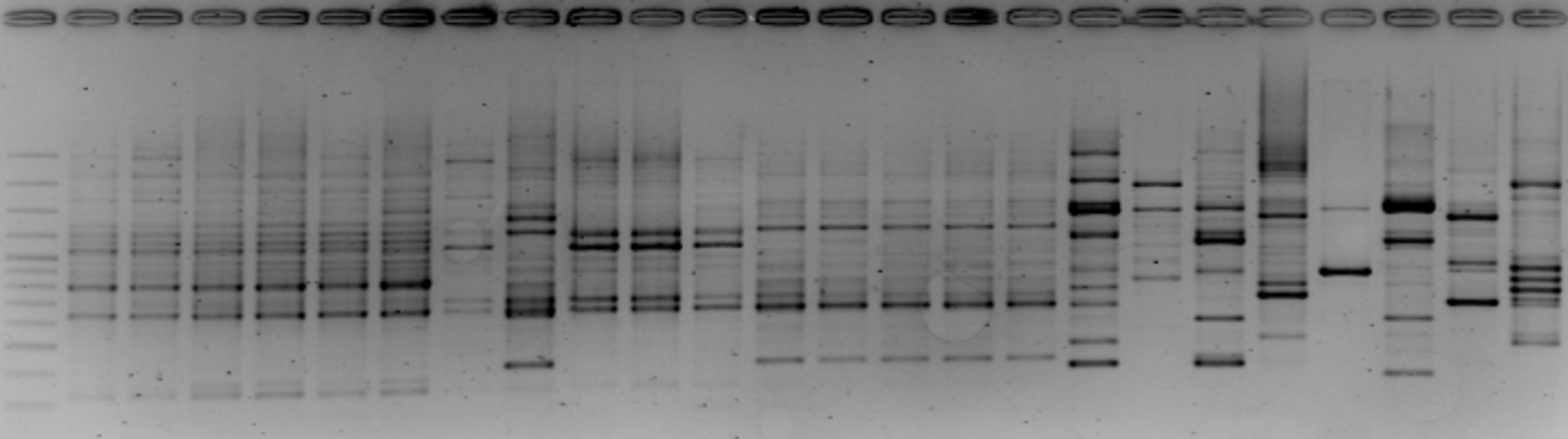

Supplement: Additional file 13: Figure S13 — SCoT profile generated from Solanum species. Bands generated with primer SCoT36 (5’-GCAACAATGGCTACCACC-3’) and separated on 1.5% agarose gel. Fingerprints are shown as a negative image of the ethidium-bromide stained gel. [file 1746-4811-9-6-S13.pdf]
