# Supplementary material for: Advances in plant gene-targeted and functional markers: a review
Source: Plant Methods. 2013 Feb 13;9:6. doi: 10.1186/1746-4811-9-6 (PMC3583794; doi:10.1186/1746-4811-9-6)
Supplement: Additional file 3: Table S1 — Major application areas of gene-targeting and functional markers discussed in the study. [file 1746-4811-9-6-S3.pdf]

Table S1. Major application areas of gene-targeting and functional markers discussed in the study.

Major applications	Marker technique	Genus	Reference
**Diversity analysis**	CDDP	*Oryza* L.	Collard and Mackill (2009a)
		Solanum L.	Poczai et al. (2011)
	PBA	*Musa* L.	Wan et al. (2005)
		Withania Pauquy	Gilani et al. (2010)
		Eleusine Gaertn.	Panwar et al. (2010)
		Aeluropus Trin.	Ahmed et al. (2011)
		Curcuma L.	Jatoi et al. (2010)
	TBP	*Brassica* L.	Bardini et al. (2004)
		Coffea L.	
		Lotus L.	
		Eleusine Gaertn.	Breviario et al. (2007)
		Arachis L.	
	ITP	*Solanum* L.	Poczai et al. (2010)
		Secale L.	Rafalski et al (2002)
		Zea L.	Holland et al. (2001)
		Hevea Aubl.	Li et al. (2012)
	IRAP/REMAP	*Pisum* L.	Pearce et al. (2000)
		Phaseolus L.	Métais et al. (2000)
		Triticum L.	Carvalho et al. (2010)
	ISAP	*Solanum* L.	Seibt et al. (2012)
	iPBS	*Linum* L.	Smýkal et al. (2011)
	SSAP	*Pisum* L.	Pearce et al. (2000)
		Phragmites Adans.	Li et al. (2009)
		Vitis L.	Labra et al. (2008)
	RGAP	*Triticum* L.	Sela et al. (2009)
		Solanum L.	Zhuang et al. (2012)
	NBS-profiling	*Triticum* L.	Mntovani et al. (2006)
		Zingiberaceae	Joshi et al. (2012)
		Cucumis L.	Goryunova et al. (2011)
	cDNA-RFLP	*Pisum* L.	Lu et al. (1996)
	EST-SSR	*Hordeum* L.	Varshney et al. (2007)
		Festuca L.	Saha et al. (2004)
		Jatropha L.	Wen et al. (2010)
		Asparagus L.	Caruso et al. (2008)
	DALP	*Stephania* Lour.	Ma et al. (2008)
		Psammosilene W.C.Wu & C.Y.Wu	Qu et al. (2010)
	PAAP	*Capsicum* L.	Ince et al. (2010)
		Gossypium L.	Pang et al. (2009)
	SRAP	*Cucurbita* L.	Ferriol et al. (2003)
		Vigna Savi	Aneja (2010)
		Morus L.	Zhao et al. (2009)
	TRAP	*Spinacia* L.	Hu et al. (2007)
		Lactuca L.	Hu et al. (2005)
		Helianthus L.	Yue et al. (2009)
	CoRAP	Salvia L.	Wang et al. (2009)

	SCoT	*Solanum* L.	Gorji et al. (2011)
		Mangifera L.	Luo et al. (2010)
		Vitis L.	Guo et al. (2012)
**Molecular identification**	TBP	*Triticum* L.	Casazza et al. (2012)
	IRAP/REMAP	*Vitis* L.	D'Onofrio et al. (2010)
		Diospyros L.	Du et al. (2009)
	iPBS	*Prunus* L.	Baránek et al. (2012)
	SSAP	*Vitis* L.	Castro et al. (2012)
		Eryngium L.	Ievina et al. (2010)
	RGAP	*Nicotiana* L.	Leng et al. (2010)
	NBS-profiling	*Lupinus* L.	You et al. (2005)
	EST-SSR	*Coffea* L.	Aggarwal et al. (2007)
		Triticum L.	Leigh et al. (2003)
		Citrus L.	Rao et al. (2008)
	DALP	*Panax* L.	Ha et al. (2001)
	TRAP	*Paeonia* L.	Zhang et al. (2012)
**Hybrid identification**	TBP	*Centaurea* L.	Ferriol et al. (2011)
	ITP	*Rhododendron* L.	Wei et al. (2006)
	IRAP/REMAP	*Solanum* L.	Lightbourn et al. (2007)
	SSAP	*Pisum* L.	Vershinin et al. (2003)
		Spartina Schreb.	Parisod et al. (2009)
		Nicotiana L.	Petit et al. (2010)
		Arabidopsis (DC.)Heynh.	Beaulieu et al. (2009)
	NBS-profiling	*Vitis* L.	Mahanil et al. (2007)
	EST-SSR	*Thinopyrum* Á.Löve	Wang et al. (2010)
		Fraxinus L.	Gérard et al. (2012)
		Helianthus L.	Ellis et al. (2006)
	SRAP	*Paeonia* L.	Hao et al. (2008)
**Marker-trait association**	PBA	*Zingiber* Mill.	Wicaksana et al. (2011)
	ITP	*Phaseolus* L.	Acampora et al. (2007)
	iPBS	*Prunus* L.	Mészáros et al. (2011)
	SSAP	*Capsicum* L./ *Solanum* L.	Tam et al. (2005)
		Brassica L.	Zou et al. (2011)
		Vicia L.	Ouji et al. (2012)
	RGAP	*Saccharum* L.	Jayashree et al. (2010)
		Triticum L.	Dong et al. (2009)
		Vitis L.	Dry et al. (2010)
	NBS-profiling	*Vicia* L.	Torres et al. (2010)
		Allium L.	Shemesh et al. (2008)
		Triticum L.	Jing et al. (2007)
	EST-SSR	*Triticum* L.	Breseghello and Sorrells (2006)
		Lactuca L.	Simko (2009)
	SRAP	*Hippophae* L.	Li et al. (2010)
	TRAP	*Caladium* Vent.	Deng et al. (2007)
		Saccharum L.	Creste et al. (2010)
	SCoT	*Arachis* L.	Xiong et al. (2011)
**Phylogenetics and**	PBA	*Tinospora* Miers.	Ahmad et al. (2009)

**systematics**			
	ITP	*Manihot* Mill.	Olsen and Schaal (1999)
		Rhododendron L.	Wei et al. (2006)
	iPBS	*Cicer* L.	Andeden et al. (2012)
	SSAP	*Triticum* L.	Gribbon et al. (1999)
		Iris L.	Cornman and Arnold (2009)
		Fragaria L.	He et al. (2012)
	RGAP	*Prunus* L.	Cao et al. (2011)
		Solanum L.	Liao et al. (2012)
	NBS-profiling	*Solanum* L.	Wang et al. (2008)
	EST-SSR	*Lolium* L./*Festuca* L.	Mian et al. (2005)
		Bambusoideae	Barkley et al. (2005)
		Crotalaria L.	Wang et al. (2006)
	SRAP	*Hedychium* J.Koenig	Gao et al. (2008)
		Pistacia L.	Talebi et al. (2012)
	TRAP	*Pelargonium* L'Hér.	Palumbo et al. (2007)
		Porphyra Lour.	Qiao et al. (2007)
	SCoT	*Solanum* L.	Poczai and Hyvönen (2011)
		Cicer L.	Amirmoradi et al. (2012)
**Genetic and comparative mapping**	PBA	*Solanum* L.	Yamanaka et al. (2005)
	ITP	*Gossypium* L.	Lin et al. (2010)
		Eragrostis Wolf	Yu et al. (2006)
		Cicer L.	Gujaria et al. (2011)
		Medicago L.	Choi et al. (2004)
	IRAP/REMAP	*Hordeum* L.	Manninen et al. (2000)
		Aegilops L.	Boyko et al. (2002)
		Citrus L.	Biswas et al. (2010)
	iPBS	*Gentiana* L.	Nakatsuka et al. (2012)
	SSAP	*Pisum* L.	Ellis et al. (1998)
		Avena L.	Yu and Wise (2000)
	RGAP	*Solanum* L.	Lebeau et al. (2011)
		Musa L.	Miller et al. (2008)
		Citrus L.	Gulsen et al. (2010)
	NBS-profiling	*Lactuca* L.	Syed et al. (2006)
		Triticum L.	Lapitan et al. (2007)
	iSNAP	*Oryza* L.	Gui et al. (2011)
	cDNA-AFLP	*Manihot* Mill.	Fregene et al. (2001)
		Oryza L.	Mao et al. (2004)
	cDNA-RFLP	*Eragrostis* Wolf	Yu et al. (2006)
		Oryza L.	Ammiraju et al. (2006)
		Gossypium L.	Ulloa et al. (2002)
	EST-SSR	*Rubus* L.	Graham et al. (2004)
		Setaria P.Beauv.	Jia et al. (2007)
		Medicago L.	Sledge et al. (2005)
	DALP	*Helianthus* L.	Langar et al (2003)
	SRAP	*Cucumis* L.	Yeboah et al. (2007)
		Dendrobium Sw.	Xue et al. (2010)

		Dactylis L.	Xie et al. (2011)
	TRAP	*Phaseolus* L.	Miklas et al. (2006)
		Helianthus L.	Chen et al. (2006)
		Saccharum L.	Alwala et al. (2008)
**QTL analysis**	ITP	*Solanum* L.	Gorji et al. (2012)
	SSAP	*Solanum* L.	Meyer et al. (1998)
		Saccharum L.	Palhares et al. (2012)
		Cynara L.	Portis et al. (2009)
	RGAP	*Solanum* L.	Danan et al. (2011)
		Lolium L.	Schejbel et al. (2008)
		Gossypium L.	Niu et al. (2011)
	NBS-profiling	*Malus* Mill.	Calenge et al. (2005)
		Capsicum L.	Voorrips et al. (2004)
	cDNA-AFLP	*Glycine* Willd.	Zhang et al. (2004)
		Oryza L.	Zheng et al. (2003)
		Gossypium L.	Lacape et al. (2003)
	cDNA-RFLP	*Agrostis* L.	Chakraborty et al. (2005)
		Helianthus L.	Genzbittel et al. (1999)
		Eucalyptus L'Hér.	Morana et al. (2002)
	EST-SSR	*Poncirus* Raf.	Chen et al. (2008)
		Quercus L.	Durand et al. (2010)
		Brassica L.	Kaur et al. (2009)
	SRAP	*Brassica* L.	Sun et al. (2007)
		Gossypium L.	Wang et al. (2006)
		Dendranthema (DC.) Des Moul	Zhang et al. (2011)
	TRAP	*Triticum* L.	Li et al. (2007)
		Aegilops L.	Wang et al. (2006)
	SCoT	*Solanum* L.	Gorji et al. (2012)
**Chromosome and genome evolution**	ITP	*Silene* L.	Bergero et al. (2007)
		Triticum L.	Ishikawa et al. (2007)
	IRAP/REMAP	*Spartina* Schreb.	Baumel et al. (2002)
		Triticum L. × *Secale* L.	Bento et al. (2008)
	SSAP	*Arabidopsis* (DC.) Heynh .	Heslop-Harrison et al. (1997)
		Nicotiana L.	Parisod et al. (2012)
	RGAP	*Musa* L.	Azhar et al. (2008)
	cDNA-AFLP	*Senecio* L.	Hegarty et al. (2005)
		Tragopogon L.	Soltis et al. (2004)
		Triticum L.	Pumphrey et al. (2009)
	cDNA-RFLP	*Triticum* L.	Pont et al. (2011)
		Capsicum L.	Livingstone et al. (1999)
		Arachis L.	Kochert et al. (1996)
	EST-SSR	*Cucumis* L.	Li et al. (2011)
		Triticum L.	Bandopadhyay et al. (2011)
		Arachis L.	Moretzsohn et al. (2009)
	DALP	*Helianthus* L.	Hu (2006)
	SRAP	*Arachis* L.	Ren et al. (2010)

References

Acampora A, Ciaffi M, De Pace C, Paolacci AR, Tanzarella OA (2007) Pattern of variation for seed size traits and molecular markers in Italian germplasm of *Phaseolus coccineus* L. Euphytica 157:69-82

Aggarwal RK, Hendre PS, Varshney RK, Bhat PR, Krishnakumar V, Singh L (2007) Identification, characterization and utilization of EST-derived genic microsatellite markers from genome analyses of coffee and related species. Theor Appl Genet 114:359-372

Ahmad, SM, Hoot SB, Qazi PH, Verma V (2009) Phylogenetic patterns and genetic diversity of Indian *Tinospora* species based on chloroplast sequence data and cytochrome P450 polymorphisms. Plant Syst Evol 281:87–96

Ahmed MZ, Gilani SA, Kikuchi A, Gulzar S, Khan MA, Watanabe KN (2011) Population diveresity of *Aeluropus lagopoides*: a potential cash crop for saline land. Pak J Bot 43:595-605

Alwala S, Kimbeng CA, Veremis JC, Gravois KA (2008) Linkage mapping and genome analysis in a *Saccharum* interspecific cross using AFLP, SRAP and TRAP markers. Euphytica 164:37-51

Amirmoradi B, Talebi R, Karami E (2012) Comparison of genetic variation and differentiation among annual *Cicer* species using start codon targeted (SCoT) polymorphism, DAMD-PCR, and ISSR markers. Plant Syst Evol 298:1679-1688

Ammiraju JSS, Luo M, Goicoechea JL, Wang W, Kudrna D, Mueller C, Talag J, Kim HR, Sisneros NB, Blackmon B et al. (2005) The Oryza bacterial artificial chromosome library resource: conctruction and analysis of 12-deep-coverage large-insert BAC libraries that represent the 10 genome types of the genus *Oryza*. Genome Res 16:140-147

Andeden EE, Baloch FS, Derya M, Kilian B, Özkan H (2012) iPBS-retrotransposons-based genetic diversity and realationships among wild annual *Cicer* species. J Plant Biochem Biotech DOI 10.1007/s13562-012-0175-5

Aneja B (2010) Micronutrient and molecular diversity analysis in mungbean [*Vigna radiata* (L.) Wilczek] genotypes. MSc thesis, CCSHAU Hisar

Azhar M, Heslop-Herrison JS (2008) Genomes, diversity and resistance gene analogues in *Musa* species. Cytogenet Genome Res 121:59-66

Bandopadhyay R, Sharma S, Rustgi S, Singh R, Kumar A, Balyan HS, Gupta PK (2004) DNA polymorphism among 18 species of *Triticum-Aegilops* compley using wheat EST-SSRs. Plant Sci 166:349-356

Baradini M, Lee D, Donini P, Mariani A, Gianí, Toschi M, Lowe C, Breviario D (2004) Tubulin-based polymorphism (TBP): a new tool, based on functionally relevant sequences, to assess genetic diversity in plant species. Genome 47:281-291

Baránek M, Mészáros M, Sochorová J, Čechová J, Raddová J (2012) Utility of retrotransposon-derived markers systems for differentiation of persumed clones of the apricot cultivar Velkopavlovická. Sci Hort 143:1-6

Barkley NA, Newman ML, Wang ML, Hotchkiss MW, Pederson GA (2005) Assessment of the genetic diversity and phylogenetic relationships of a temperate bamboo collection by using transferred EST-SSR markers. Genome 48:731-737

Baumel A, Ainouche M, Kalendar R, Schulman AH (2002) Retrotransposons and genomic stability in populations of the young allopolyploid species *Spartina anglica* C.E. Hubbard (Poaceae). Mol Biol Evol 19:1218-1227

Beaulieu J, Jean M, Belzile F (2009) The allotetraploid *Arabidopsis thaliana – Arabidopsis lyrata* subsp. *petraea* as an alternative model system for the study of polypolidy in plants. Mol Gen Genom 281:421-435

Bento M, Pereira HS, Rochetra M, Gustafson P, Viegas W, Silva M (2008) Polyploidization as a restriction force in plant genome evolution: sequence rearrangements in Triticale. PloS ONE 3:e1402

Bergero R, Forrest A, Kamau E, Charlesworth D (2007) Evolutionary strata on the X chromosomes of the dioecious plant *Silene latifolia*: evidence from new sex-linked genes. Genetics 175:1945-1954

Biswas MK, Xu Q, Deng X-x (2010) Utility of RAPD, ISSR, IRAP and REMAP markers for the genetic analysis of *Citrus* spp. Sci Hort 124:254-261

Boyko E, Kalendar R, Korzun V, Fellers J, Korol A, Schulman AH, Gill BS (2002) A high-density cytogenetic map of the *Aegilops tauschii* genome incorporating retrotransposons and degense-related genes: insights into cereal chromosome strucutre and function. Plan Mol Biol 48:767-789

Breseghello F, Sorrells ME (2006) Association mapping of kernel size and milling quality in wheat (*Triticum aestivum* L.) cultivars. Genetics 172:1165-1177

Breviario D, Baird WmV, Sangoi S, Hilu K, Blumetti P, Gianí S (2007) High polymorphism and resolution in targeted fingerprinting with combined β-tubulin introns. Mol Breeding 20:249-259

Calenge F, van der Linden CG, van de Weg E, Schouten HJ, van Arkel G, Denancé C, Durel C-E (2005) Resistance gene analogues identified through the NBS-profiling methods map close to the major genes and QTL for disease resistance in apple. Theor Appl Genet 110:660-668

Cao K, Wang LR, Zhu GR, Fang WCH, Chen CHW (2011) Isolation, characterization and phylogenetic analysis of resistance gene analogues in a wild species of peach (*Prunus kansuensis*). Can J Plant Sci 91:961-970

Caruso M, Federici CT, Roose ML (2008) EST-SSR markers for asparagus genetic diversity evaluation and cultivar identification. Mol Breeding 21:195-204

Carvalho A, Guedes-Pinto H, Martins-Lopes P, Lima-Brito J (2010) Genetic variability of Old Portugese bread wheat cultivars assayed by IRAP and REMAP markers. Ann Appl Biol 156:337-345

Casazza AP, Morcia C, Ponzoni E, Gavazzi F, Bendettelli S, Breviario D (2012) A reliable assay for the detection of soft wheat adulteration in Italian pasta is based on the use of new DNA molecular markers capable of discriminating between *Triticum aestivum* and *Triticum durum*. J Cereal Sci 56:733-740

Castro I, D’Onofrio C, Martín JP, Ortiz JJ, De Lorenzis G, Ferreeira V, Pinto-Carnide O (2012) Effectivness of AFLPs and retrotransposon-based markers for the identification of portugese grapevine cultivars and clones. Mol Biotechnol 52:26-39

Chakraborty N, Bae J, Warnke S, Chang T, Jung G (2005) Linkage map construction in allotetrapolid creeping bentgrass (*Agrostis stolonifera* L.). Theor Appl Genet 111:795-803

Chen C, Bowman KD, Choi YA, Dang PM, Rao MN, Huang S, Soneji JR, McCollum TG, Gmitter FG, Jr (2008) EST-SSR genetic maps for *Citrus sinensis* and *Poncirus trifoliata*. Tree Gen Genoms 4:1-10

Chen J, Hu J, Vick BA, Jan CC (2006) Molecular mapping of a nuclear male-sterility gene in sunflower (*Helianthus annuus* L.) using TRAP and SSR markers. Theor Appl Genet 113:122-127

Choi H-K, Kim D, Uhm T, Limpers E, Lim H, Mun J-H, Kalo P, Penmesta RV, Seres A, Kulikova O et al. (2004) A sequence-based genetic map of *Medicago truncatula* and comparison of marker colineearity with *M. sativa*. Genetics 166:1463-1502

Collard BCY, Mackill DJ (2009) Conserved DNA-derived polymorphism (CDDP): a simple and novel methods for generating DNA markers in plants. Plant Mol Biol Rep 27:558-562

Cornman RS, Arnold ML (2009) Characterization and comparative analysis of sequence-specific amplified polymorphisms based on two subfamilies of IRRE retrotransposons in *Iris missouriensis* (Iridaceae). Genetica 135:25-38

Creste S, Accoroni KAG, Pinto LR, Vencovsky R, Gimenes MA, Xavier MA, Landell MGA (2010) Genetic variability among sugarcane genotypes based on polymorphisms in sucrose metabolism and drought tolerance genes. Euphytica 172:435-446

D'Onofrio C, De Lorenzis G, Giordani T, Natali L, Cavallini A, Scalabrelli G (2010) Retrotransposon-based molecular markers for grape species and cultivars identification. Tree Gen Genomes 6:451-466

Danan S, Veyrieras J-B, Lefebvre V (2011) Construction of a potato consensus map and QTL meta-analysis offer new insights into the genetic architecture of late blight resistance and plant maturity traits. BMC Plant Biol 11:16

Deng Z, Goktepe F, Harbaugh BK, Hu J (2007) Assessment of genetic diversity and relationships among *Caladium* cultivars and species using molecular markers. J Am Soc Hort Sci 132:219-229

Dong P, Wei Y-M, Chen G-Y, Li W, Nevo E, Zheng Y-L (2009) Resistance gene analog polymorphisms (RGAPs) in wild emmer wheat (*Triticum dicoccoides*) and their ecological associations. 56:121-136

Dry IB, Feechan A, Anderson C, Jermakow AM, Bouquet A, Adam-Blondon A-F, Thomas MR (2010) Molecular strategies to enchance the genetic resistance of grapevines to powdery mildew. Aust J Grape Wine R 16 (s1):94-105

Du XY, Zhang QL, Luo Z-R (2009) Comparision of four molecular markers for genetic analysis in *Diospyros* L. (Ebenaceae). Plant Syst Evol 281:171-181

Durand J, Bodénés C, Chancerel E, Frigerio J-M, Vendramin G, Sebastiani F, Bounamici A, Gailang O, Koelewijn H-P, Villani F et al. (2010) A fast and cost-effective approach to develop and map EST-SSR markers: oak as a case study. BMC Genomics 11:570

Ellis JR, Pashley CH, Burke JM, McCauley DE (2006) High genetic diversity in a rare and endangered sunflower as compared to a common congener. Mol Ecol 15:2345-2355

Ellis THN, Poyser SJ, Knox MR, Vershinin AV, Ambrose MJ (1998) Polymorphsim of insertion sites of *Ty1-copia* class retrotransposons and its use for linkage and diversity analysis in pea. Mol Gen Genet 260:9-19

Ferriol M, Garmendia A, Ruiz JJ, Merle H, Boira H (2012) Morphological and molecular analysis of natural hybrids between the diploid *Centaurea aspera* L. and the tetraploid *C. seridis* L. (Compositae). Plant Biosyst 146:86-100

Ferriol M, Picó B, Nuez F (2003) Genetic diversity of a germplasm collection of *Cucurbita pepo* using SRAP and AFLP markers. Theor Appl Genet 107:271-282

Fregene M, Okogbenin E, Mba C, Angel F, Suarez MC, Janneth G, Chavarriaga P, Roca W, Bonierbale M, Tohme J (2001) Genome mapping in cassava improvement: challanges, achievments and opportunitites. Euphytica 120:159-165

Gao L, Liu N, Huang B, Hu X (2008) Phylogenetic analysis and genetic mapping of Chinese *Hedychium* using SRAP markeres. Sci Hort 117:369-377

Gentzbittel L, Mestries E, Mouzeyar S, Mazeyrat F, Badaoui S, Vear F, Tourvieille de Labrouche D, Nicolas P (1999) A composite map of expressed sequences and phenotypic traits of the sunflower (*Helinathus annuus* L.) genome. Theor Appl Genet 99:218-234

Gérard PR, Temunović M, Sannier J, Bertolino P, Dufour J, Frascaria-Lacoste N, Fernández-Manjarrés JF (2012) Chilled but not frosty: understanding the role of climate in the hybridization between the Mediterranean *Fraxinus angustifolia* Vahl and the temperate *Fraxinus excelsior* L. (Oleaceae) ash trees. J Biogeography DOI 10.1111/jbi.12021

Gilani SA, Kikuchi A, Watanabe KN (2009) Genetic variation within and among gragmented populations of endangered medicinal plant, *Withania coagulans* (Solanaceae) from Pakistan and its implications for conservation. Afr J Biotech 8:2948-2958

Gorji AM, Mátyás KK, Dublecz Z, Decsi K, Cenrák I, Hoffmann B, Taller J, Polgár Z (2012) *In vitro* osmotic stress tolerance in potato and identification of major QTLs. Am J Pot Res 89:453-464

Gorji AM, Poczai P, Polgár Z, Taller J (2011) Efficiency of arbitrarily amplified dominant markers (SCoT, ISSR and RAPD) for diagnostic fingerprinting in tertapolid potato. Am J Pot Res 88:226-237

Goryunova SV, Gashkova IV, Kosareva GA (2011) Variability and phylogenetic relationships of the *Cucumis sativus* L. species inferred from NBS-profiling and RAPD analysis. Rus J Genet 47:931-941

Graham J, Smith K, MacKenzie K, Jorgenson L, Hackett C, Powell W (2004) The construction of a genetic linkage map of red raspberry (*Rubus idaeus* subsp. *idaeus*) based on AFLPs, genomic-SSR and EST-SSR markers. Theor Appl Genet 109:740-749

Gribbon BM, Pearce SR, Kalendar R, Schulman AH, Paulin L, Jack P, Kumar A, Flavell AJ (1999) Phylogeny and transpositional activity of Ty1-*copia* group retrotransposons in cereal genomes. Mol Gen Genomes 261:883-891

Gui Y, Yan G, Bo S, Tong Z, Wang Y, Xiao B, Lu X, Li Y, Wu W, Fan L (2011) iSNAP: a small RNA-based molecular marker technique. Plant Breeding 130:515-520

Gujaria N, Kumar A, Dauthal P, Dubey A, Hiremath P, Prakash AB, Farmer A, Bhide M, Shah T, Gaur PM et al. (2011) Development and use of genic molecular markers (GMMs) for construction of a transcript map of chickpea (*Cicer arietinum* L.). Theor Appl Genet 122:1577-1589

Gulsen O, Uzun A, Canan I, Seday U, Canihos E (2010) A new citrus linkage map based on SRAP, SSR, ISSR, POGP, RGA and RAPD markers. Euphytica 173:265-277

Guo D-L, Zhang J-Y, Liu C-H (2012) Genetic diversity in some grape varieties revealed by SCoT analysis. Mol Biol Rep 39:5207-5313

Ha W-Y, Yau FC-F, But PP-H, Wang J, Shaw P-C (2001) Direct amplification of length polymorphism analysis differentiates *Panax ginseng* from *P. quinquefolius*. Planta Med 67:587-589

Hao Q, Liu Z-A, Shu Q-Y, Zhang R, De Rick J, Wang L-S (2008) Studies on *Paeonia* cultivars and hybrids identifaction based on SRAP analysis. Hereditas 145:38-47

He P, Ma Y, Dai H, Li L, Liu Y, Li H, Zhao G, Zhang Z (2012) Develpoment of Ty1-copia retrotransposon-based S-SAP markers in strawberry (*Fragaria* × *ananassa* Duch.) Sci Hort 137:43-48

Hegarty MJ, Jones JM, Wilson ID, Barker GL, Coghill JA, Sanchez-Baracaldo P, Liu G, Buggs RJA, Abbott RJ, Edwards KJ, Hiscock SJ (2005) Development of anonymous cDNA microarrays to study cahnges to the *Senecio* floral transcriptome during hybrid speciation. Mol Ecol 14:2493-2510

Heslop-Harrison JS, Brandes A, Taketa S, Scmidt T, Vershinin AV, Alkimova EG, Kamm A, Doudrick RL, Schwarzacher T, Katsiotis A et al. (1997) The chromosomal distribution of Ty1-copia group retrotransposable elements in higher plants and their implications for genome evolution. Genetica 100:197-204

Holland JB, Helland SJ, Sharapova N, Rhyne DC (2001) Polymorphism of PCR-based markers targeting exons, introns, promoter regions, and SSRs in maize and intron and repeat sequences in oat. Genome 44:1065-1076

Hu J (2006) Defining the sunflower (*Helianthus annuus* L.) linkage group ends with the *Arabidopsis*-type telomere sequence repeat-derived markers. Chromosome Res 14:535-548

Hu J, Mou B, Vick BA (2007) Genetic diversity of 38 spinach (*Spinacia oleracea* L.) germplasm accessions and 10 commercial hybrids assessed by TRAP markers. Gen Res Crop Evol 54:1667-1667

Hu J, Ochoa OE, Truco MJ, Vick BA (2005) Application of the TRAP technique to lettuce (*Lactuca sativa* L.) genotyping. Euphytica 144:225-235

Ievina B, Syed NH, Flavell AJ, Ievinsh G, Rostocks N (2010) Development of retrotransposon-based SSAP molecular marker system for study of genetic diversity in sea holly (*Eryngium maritimum* L.) Plant Genet Res 8:258-266

Ince AG, Karaca M, Onus AN (2010) Genetic relationships within and between *Capsicum* species. Biochem Genet 48:83-95

Ishikawa G, Yonemaru J, Saito M, Nakamura T (2007) PCR-based landmark unique gene (PLUG) markers effectively assign homoelogous wheat genes to A, B and D genomes. BMC Genomics 8:135

Jatoi SA, Kikuchi A, Ahmad D, Watanabe KN (2010) Characterization of the genetic strucutre of mango ginger (Curcuma amada Roxb.) from Myanmar in farm and genebank collection by the neutral and funcional genomic markers. Electron J Biotechnol 13: http://dx.doi.org/10.2225/vol13-issue6-fulltext-10

Jayashree J, Selvi A, Nair NV (2010) Characterization of resistance gene analog polymorphism in sugarcane cultivars with varying levels of red rot resistance. Elect J Plant Breeding 1:1191-1199

Jia X-P, Shi Y-S, Song Y-C, Wang G-Y, Wang T-Y, Li Y (2007) Development of EST-SSR in foxtail millet (*Setaria italica*). Gen Res Crop Evol 54:233-236

Jing H-C, Kornyukhin D, Kanyuka K, Orford S, Zlatska A, Mitrofanova OP, Koebner R, Hammond-Kosack K (2007) Identification of variation in daptively important traits and genome-wide analysis of trait-marker associations in *Triticum monococcum*. J Exp Bot 58:3749-3764

Joshi RK, Mohanty S, Kar B, Nayak S (2012) Assessment of genetic diveresity in Zingiberaceae through nucleotide binding site-based motif-directed profiling. Biochem Genet 50:642-656

Kaur S, Cogan NOI, Ye G, Baillie RC, Hand ML, Ling AE, Mcgearey AK, Kaur J, Hopkins CJ, Todorovic M et al. (2009) Genetic map construction and QTL mapping of resistance to blackleg (*Leptosphaeria maculans*) disease in Australian canola (*Brassica napus* L.) cultivars. Theor Appl Genet 120:71-83

Kochert G, Stalker HT, Gimenes M, Galgaro L, Lopes CR, Moore K (1996) RFLP and cytogenetic evidence on the origin and evolution of allotetraploid domesticated peanut, *Arachis hypogaea* (Leguminosae). Am J Bot 83:1282-1291

Labra M, Imazio S, Grassi F, Rossoni M, Sala F (2004) *Vine-1* retrotransposon-based sequence-specific amplified polymorphism for *Vitis vinifera* L. genotyping. Plant Breeding 123:180-185

Lacape J-M, Nguyen T-B, Thibivilliers S, Bojonov B, Courtiois B, Cantrell RG, Burr B, Hau B (2003) A combined RFLP, SSR, AFLP map of tetrapolid cotton based on a *Gossypium hirsutum* × *Gossypium barbadebse* backcross population. Genome 46:612-626

Langar K, Lorieux M, Desmarais E, Griveau Y, Gentzbittel L, Bervillé A (2003) Combined mapping of DALP and AFLP markers in cultivated sunflower using F9 recombinanat inbred lines. Theor Appl Genet 106:1068-1074

Lapitan NLV, Peng J, Sharma V (2006) A high-density map and PCR markers for Russian wheat aphid resistance gene Dn7 on chromosome 1RS/1BL. Crop Sci 47:811-818

Lebeau A, Gouy M, Daunay MC, Wicker E, Chiroleu F, Prior P, Frary A, Dintinger J (2012) Genetic mapping of a major dominanat gene for resistance to *Ralstonia solanacearum* in eggplant. Theor Appl Genet DOI 10.1007/s00122-012-1969-5

Leigh F, Lea V, Law J, Wolters P, Powell W, Donini P (2003) Assessment of EST- and genomic microsatellite markers for variety discrimination and genetic diversity studies in wheat. Euphytica 133:359-366

Leng X, Xiao B, Wang S, Gui Y, Wang Y, Lu X, Xie J, Li Y, Fan L (2010) Identification of NBS-type resistance gene homologs in tobacco genome. Plant Mol Biol Rep 28:152-161

Li D, Cuevas HE, Yang L, Li Y, Garcia-Mas J, Zalpa J, Staub JE, Luan F, Reddy U, Luan F et al. (2011) Syntenic relationships between cucumber (*Cucumis sativus* L.) and melon (*C.melo* L.) chromosomes as revealed by comparative genetic mapping. BMC Genomics 12:396

Li D, Xia Z, Deng Z, Liu X, Dong J, Feng F (2012) Development and characterization of intron-flanking EST-PCR markers in rubber tree (*Hevea brasiliensis* Muell.Arg.). Mol Biotechnol 51:148-159

Li H, Ruan C-J, Teixeira da Silva JA, Liu B-Q (2010) Association of SRAP markers with dried-shirnk disease resistance in a germplasm collection of sea buckhorn (*Hippophae* L.). Genome 53:447-457

Li M, Gong L, Tian Q, Hu L, Guo W, Kimatu JN, Wang D, Liu B (2009) Clonal genetic diversity and populational genetic differentiation in *Phramites australis* distributed in the Songnen Praire in northeast China as revealed by amplified fragment length polymorphism and sequence-specific polymorphism molecular markers. Ann Appl Biol 154:43-55

Li S, Jia J, Wei X, Zhang X, Li L, Chen H, Fan Y, Sun H, Zhao X, Lei T et al. (2007) A intervarietal genetic map and QTL analysis for yield traits in wheat. Mol Breeding 20:167-178

Liao P-C, Lin K-H, Ko C-L, Hwang S-Y (2011) Molecular evolution of a family of resistance gene analogs of nucleotide-binding site sequences in *Solanum lycopersicum*. Genetica 139:1229-1240

Lightbourn GJ, Jelesko JG, Veilleux RE (2007) Retrotransposon-based markers from potato monoploids used in somatic hybridization. Genome 50:492-501

Lin L, Pierce GJ, Bowers JE, Estill JC, Compton RO, Rainville LK, Kim C, Lemeke C, Rong J, Tang H et al. (2010) A draft physical map of a D-genome cotton species (*Gossypium raimondii*). BMC Genomics 11:395

Livingstone KD, Lackney VK, Blauth JR, van Wijk R, Jahn MK (1999) Genome mapping in *Capsicum* and the evolution of genome strucutre in the Solanaceae. Genetics 152:1183-1202

Lu J, Lnox MR, Ambrose MJ, Brown JKM, Ellis THN (1996) Comparative analysis of genetic diversity in pea assessed by RFLP-and PCR-based methods. Theor Appl Genet 93:1103-1111

Luo C, He X-H, Chen H, Ou S-J, Gao M-P (2010) Analysis of diversity and relationships among mango cultivars using Start Codon Targeted (SCoT) markers. Biochem Syst Ecol 38:1176-1184

Ma Y-s, Yu H, Li Y-y, Yan H, Cheng X (2008) A study of genetic strucutre of *Stephania yunnanensis* (Menispermaceae) by DALP. Biochem Genet 46:227-240

Mahanil S, Reisch BI, Owens CL, Thipyapong P, Laosuwan P (2007) Resistance gene analogs from *Vitis cinerea*, *Vitis rupestirs*, and *Vitis* hybrid Horizon. Am J Enol Vitic 58:484-493

Mannien O, Kalendar R, Robinson J, Schulman AH (2000) Application of BARE-1 retrotransposon markers to the mapping of a major resistance gene for net blotch in barley. Mol Gen Genet 264:325-334

Mantovani P, van der Linden G, Maccaferri M, Sanguineti MC, Tuberosa R (2006) Nucleotide-binding site (NBS) profiling of genetic diversity in durum wheat. Genome 49:1473-1480

Mao C, Yi K, Yang L, Zheng B, Wu Y, Liu F, Wu P (2004) Identification of aluminium-regulated genes by cDNA-AFLP in rice (*Oryza sativa* L.): aluminium-regulated genes for the metabolism of cell wall components. J Exp Bot 55:137-143

Mészáros M, Krška B, Baránek M, Radová J (2011) Relation of floral morphological characters to genetic variation at transposon level of Velkopavlovická cultivar apricot clones. Acta Agr Serb 31:59-72

Métais I, Aubury C, Hamon B, Jalouzot R, Peltier D (2000) Description and analysis of genetic diversity between commercial bean lines (*Phaseolus vulgaris* L.). Theor Appl Genet 101:1207-1214

Meyer RC, Milbourne D, Hackett CA, Bradshaw JE, McNichol JW, Waugh R (1998) Linkage analysis in tetrapolid potato and association of markers with quantitative resistance to late blight (*Phytophthora infestans*). Mol Gen Genet 259:150-160

Mian MAR, Saha MC, Hopkins AA, Wang Z-Y (2005) Use of tall fescue EST-SSR markers in phylogenetic analysis of cool-season forage grasses. Genome 48:637-647

Miklas PN, Hu J, Grünwald NJ, Larsen KM (2006) Potential application of TRAP (Targeted Region Amplified Polymorphism) markers for mapping and tagging disease resistance traits in common bean. Crop Sci 46:910-916

Miller RNG, Bertioli DJ, Baurens FC, Santos CMR, Alves PC, Martins NF, Togawa RC, Souza MT, Pappas GJ (2008) Analysis of non-TIR NBS-LRR resistance gene analogs in *Musa acuminata* Colla: isolation, RFLP marker development, and physical mapping. BMC Plant Biol 8:15

Moran GF, Thamarus KA, Raymond CA, Qiu D, Uren T, Southerton SG (2002) Genomics of *Eucalyptus* wood traits. Ann For Sci 59:645-650

Moretzsohn MC, Barbosa AVG, Alves-Freitas DMT, Teixeria C, Leal-Bertioli CM, Guimarães PM, Pereira RW, Lopes CR, Cavallari MM, Valls JFM et al. (2009) A linkage map for the B-genome of *Arachis* (Fabaceae) and its synteny to the A-genome. BMC Plant Biol 9:40

Nakatsuka T, Yamada E, Saito M, Hikage T, Ushiku Y, Nishihara M (2012) Construction of the first genetic linkage map of Japanese gentian (Gentianaceae). BMC Genomics 13:672

Niu C, Lu Y, Yuan Y, Percy RG, Ulloa M, Zhang J (2011) Mapping resistance gene analogs (RGAs) in cultivated tetrapolid cotton using RGA-AFLP analysis. Euphytica 181:65-76

Olsen KM, Schaal BA (1999) Evidence on the origin of cassava: phylogeography of *Manihot esculenta*. PNAS 96:5586-5591

Ouji A, El Bok S, Syed NH, Abdellaoui R, Rouaissi M, Flavell AJ, El Gazzah M (2012) Genetic diversity of faba bean (*Vicia faba* L.) populations revealed by sequence specific amplified polymorphsim (SSAP) markers. Afr J Biotech 11:2162-2168

Palhares AC, Rodrigues-Morais TB, Van Sluys M-A, Domingues DS, Maccheroni W, Jordão H, Souza AP, Marconi TG, Mollinari M, Gazaffi R et al. (2012) A novel linkage map of sugercane with evidence for clustering of retrotransposon-based markers. BMC Genet 13:51

Palumbo R, Hong W-F, Wang G-L, Hu J, Craig R, Locke J, Krause C, Tay D (2007) Target region amplification polymorphism (TRAP) as a tool for detecting genetic variation in the genus *Pelargonium*. Hort Sci 42:1118-1123

Pang M, Percy RG, Hughs E, Zhang J (2009) Promoter anchored amplified polymorphism based on random amplified polymorphic DNA (PAAP-RAPD) in cotton. Euphytica 167:281-291

Panwar P, Nath M, Yadav VK, Kumar A (2010) Comparative evaluation of genetic diversity using RAPD, SSR and cytochrome P450 gene based markers with respect to calcium content in finger millet (*Eleusine corcana* L. Gartn.). J Genet 89:121-133

Parisod C, Mhiri C, Lim KY, Clarkson JJ, Chase MW, Leitch AR, Grandbastien M-A (2012) Differential dynamics of transposable elements during long-term diploidization of Nicotiana section Repandae (Solanaceae) allopolyploid genomes. PloS ONE 7:e50352

Parisod C, Salmon A, Zerjal T, Tenaillon M, Grandbastien MA, Ainouche ML (2009) Rapid structural and epigenetic reorganization near transposable elements in hybrid and allopolyploid genomes in *Spartina*. New Phytol 184: 1003–1015

Pearce SR, Knox M, Ellis THN, Flavell AJ, Kumar A (2000) Pea Ty1-*copia* group retrotransposons: transcriptional activity and use as markers to study genetic diversity in *Pisum*. Mol Gen Genet 263:898-907

Petit M, Guidat C, Daniel J, Denis E, Montoriol E, Bui QT, Lim KY, Kovarik A, Leitch AR, Grandbastien M-A, Mhiri C (2010) Mobilization of retrotransposons in synthetic allotetraploid tobacco. New Phytol 186:135-147

Poczai P, Cernék I, Gorji AM, Nagy S, Taller J, Polgár Z (2010) Develpoment of intron targeting (IT) markers for potato and cross-species amplification in *Solanum nigrum* (Solanaceae). Am J Bot 97:e142-e145

Poczai P, Hyvönen J (2011) On the origin of *Solanum nigrum*: can networks help? Mol Biol Rep 38:1171-1185

Poczai P, Varga I, Bell NE, Hyvönen J (2011) Genetic diveresity assessment of bittersweet (*Solanum dulcamara*, Solanaceae) germplasm using conserved DNA-derived polymorphism and intron-targeting marjers. Ann Appl Biol 159:141-153

Pont C, Murat F, Confolent C, Balzergue S, Salse J (2011) RNA-seq in grain unveils fate of neo- and paleopolyploidization events in bread wheat (*Triticum aestivum* L.) Genome Biol 12:R119

Portis E, Mauromicale G, Mauro R, Acquadro A, Scaglione D, Lanteri S (2009) Construction of a reference molecular linkage map of globe artichoke (*Cynara cardunculus* var. *scolymus*). Theor Appl Genet 120:59-70

Pumphrey M, Bai J, Laudencia-Chingcuanco D, Anderson O, Gill BS (2008) Nonadditive expression of homoeologous genes is established upon polyploidization in hexaploid wheat. Genetics 181:1147-1157

Qiao L, Liu H, Sun J, Zhao F, Guo B, Weng M, Liu T, Dai Wang B (2007) Application of target region amplification polymorphism (TRAP) technique to *Porphyra* (Bangiales, Rhodophyta) fingerprinting. Phycologia 46:450-455

Qu Y, Yu H, Wu G, Ma R-F, Li Y-Y (2010) Genetic diversity and population strucure of the endangered species *Psammosilene tuncoides* revealed by DALP analysis. Biochem Syst Ecol 38:880-887

Rafalski A, Madej L, Wiśniewska I, Gaweł M (2002) The genetic diversity of componenets of rye hybrids. Cell Mol Biol Lett 7:471-475

Rao MN, Soneji JR, Chen C, Huang S, Gmitter FG, Jr. (2008) Characterization of zygotic and nucellar seedlings from sour orange-like citrus rootsock cndidates using RAPD and EST-SSR markers. Tree Genet Genomes 4:113-124

Ren X, Huang J, Liao B, Zhang X, Jiang H (2010) Genomic affinities of *Arachis* genus and interspecific hybrids were revealed by SRAP markers. Gen Res Crop Evol 57:903-913

Saha MC, Mian MAR, Eujayl I, Zwonitzer JC, Wang L, May GD (2004) Tall fescue EST-SSR markers with transferability across several grass species. Theor Appl Genet 109:783-791

Schejbel B, Jensen LB, Asp T, Xing Y, Lübberstedt T (2008) mapping of QTL for resistance to powdery mildew and resistance gene analogues in perennial rygrass. Plant Breeding 127:368-375

Seibt KM, Wenke T, Wollrab C, Junghans H, Muders K, Dehmer KJ, Diekmann K, Schmidt T (2012) Development and application of SINE-based markers for genotyping of potato variaites. Theor Appl Genet 125:185-196

Sela H, Cheng J, Jun Y, Nevo E, Fajima T (2009) Divergent diversity patterns of NBS and LRR domains of resitance gene analogs in wild emmer wheat populations. Genome 52:557-565

Shemesh E, Scholten O, Rabinowitch HD, Kamenetsky R (2008) Unlocking variability: inherent variation and developmental traits of garlic plants originated from sexual reproduction. Planta 227:1013-1024

Simkp I (2009) Develpoment of EST-SSR markers for the study of population strucutre in lettuce (*Lactuca sativa* L.) J Hered 100:256-262

Sledge MK, Ray IM, Jiang G (2005) An expressed sequence tag SSR map of tetraploid alfalfa (*Medicago sativa* L.) Theor Appl Genet 111:980-992

Smýkal P, Bačová-Kerteszová N, Kalendar R, Corander J, Schulman AH, Pavelek M (2011) Genetic diversity of cultivated flax (*Linum usitatissimum* L.) germplasm assessed by retrotransposon-based markers. Theor Appl Genet 122:1385-1397

Soltis DE, Soltis PS, Pires JC, Kovarik A, Tate JA, Mavrodiev E (2004) Recent and recurrent polypoloidy in *Tragopogon* (Asteraceae): cytogenetic, genomic and genetic comparisons. Biol J Linn Soc 82:485-501

Sun Z, Wang Z, Tu J, Zhang J, Yu F, McVetty PBE, Li G (2007) An ultradense genetic recombination map for *Brassica napus*, consisting of 13551 SRAP markeres. Theor Appl Genet 114:1305-1317

Syed NH, Sørensen AP, Antonise R, van de Wiel C, van der Linden CG, van't Westende W, Hooftman DAP, den Nijs HCM, Flavell AJ (2006) A detailed linkage map of lettuce based on SSAP, AFLP and NBS markers. Theor Appl Genet 112:517-527

Talebi M, Kazemi M, Sayed-Tabatabaei BE (2012) Molecular diversity and phylogenetic relationships of *Pistacia vera*, *Pistacia atlantica* subsp. *mutica* and *Pistacia khinjuk* using SRAP markers. Biochem Syst Ecol 44:179-185

Tam SM, Mhiri C, Vogelaar A, Kerkveld M, Pearce SR, Grandbastien M-A (2005) Comparative analyses of genetic diversities within tomato and pepper collections detected by ratrotransposon-based SSAP, AFLP and SSR. Theor Appl Genet 110:819-831

Torres AM, Avila CM, Gutierrez N, Palomino C, Moreno MT, Cubero JI (2010) Marker-assisted selection in faba bean (*Vicia faba* L.) Field Crop Res 115:243-252

Ulloa M, Meredith WR, Jr, Shappley ZW, Kahler AL (2002) RFLP genetic linkage maps from $F_{2.3}$ populations and a joinmap of *Gossypium hirsutum* L. Theor Appl Genet 104:200-208

Varshney RK, Chabane K, Hendre PS, Aggarwal RK, Graner A (2007) Comparative assessment of EST-SSR, EST-SNP and AFLP markers for evaluation genetic diversity and conservation of genetic resources using wild, cultivated and elite barleys. Plant Sci 173:638-6489

Vershinin AV, Allnutt TR, Knox MR, Ambrose MJ, Noel Ellis TH (2003) Transposable elements reveal the impact of introgression, rather than transposition, in *Pisum* diversity, evolution, and domestication. Mol Biol Evol 20:2067-2075

Voorrips RE, Finkers R, Sanjaya L, Groenwold R (2004) QTL mapping of anthracnose (*Colletotrichum* spp.) resistance in a cross between *Capsicum annuum* and *C. chinense*. Theor Appl Genet 109:1275-1282

Wan Y, Watanabe JA, Yi SS, Htaik T, Win K, Yamanaka S, Nakamura I, Watanabe KN (2005) Assessment of genetic diversity among the major Myanmar banana landraces. Breeding Sci 55:365-369

Wang B, Guo W, Zhu X, Wu Y, Huang N, Zhang T (2006) QTL mapping of fiber quality in an elite hybrid derived-RIL population of upland cotton. Euphytica 152:367-378

Wang M, Van den Berg R, Van der Linden G, Vosman B (2008) The utility of NBS profiling for plant systematics: a first study in tuber-bearing *Solanum* species. Plant Syst Evol 276:137-148

Wang ML, Mosjidis JA, Morris JB, Dean RE, Jenkins TM, Pederson GA (2006) Genetic diversity of *Crotalaria* germplasm assessed through phylogenetic analysis of EST-SSR markers. Genome 49:707-715

Wang Q, Thang B, Lu Q (2009) Conserved region amplified polymorphism (CoRAP), a novel marker technique for plant genotyping in *Salvia miltiorrhiza*. Plant Mol Biol Rep 27:139-143

Wang RR-C, Larson SR, Jensen KB (2010) Analyses of *Thinopyrum bessarabicum*, *T. elongatum*, and *T. junceum* chrmosomes using EST-SSR markers. Genome 53:1083-1089

Wang T, Xu SS, Harris MO, Hu J, Liu L, Cai X (2006) Genetic characterization and molecular mapping of Hessian fly resistance genes derived from *Aegilops tauschii* in synthetic wheat. Theor Appl Genet 113:611-618

Wei H, Fu Y, Arora R (2006) Utilization of intron-flanking EST-specific markers in the phylogenetic analysis and parentage identification of *Rhododendron* species and hybrids. J Am Soc Hort Sci 131:814-819

Wen M, Wang H, Xia Z, Zou M, Lu C, Wang W (2010) Development of EST-SSR and genomic-SSR markers to assess genetic diveresity in *Jatropha curcas* L. BMC Res Notes 3:42

Wickasana N, Giliani SA, Ahmad D, Kikuchi A, Watanabe KN (2011) Morphological and molecular characterization of underutilized medicinal wild ginger (*Zingiber barbatum* Wall.) from Myanmar. Plant Genet Res 9:531-542

Xie W, Zhang X, Cai H, Huang L, Peng Y, Ma X (2011) Genetic maps of SSR and SRAP markers in diploid orchardgrass (*Dactylis glomerata* L.) using the pseudo-testcriss strategy. Genome 54:212-221

Xiong F, Zhong R, Han Z, Jiang J, He L, Zhuang W, Tang R (2011) Start codon targeted polymorphism for evaluation of functional genetic variation and realationships in cultivated peanut (*Arachis hypogaea* L.) genotypes. Mol Biol Rep 38:3487-3494

Xue D, Feng S, Zhao H, Jiang H, Shen B, Shi N, Lu J, Liu J, Wang H (2010) The linkage maps of *Dendrobium* species based on RAPD and SRAP markers. J Gen Genoms 37:197-204

Yamanaka S, Ikeda S, Imai A, Luan Y, Watanabe JA, Watanabe KN (2005) Construction of integrated genetic map between various existing DNA markers and newly development P450-related PBA markers in diploid potato (*Solanum tuberosum*). Breeding Sci 55:223-230

Yeboah MA, Xuehao C, Feng CR, Liang G, Gu M (2007) A genetic linkage map of cucumber (*Cucumis sativus* L) combining SRAP and ISSR markers. Afr J Biotech 6:2784-2791

You M, Boersma JG, Buirchell BJ, Sweetingham MW, Siddique KHM, Yang H (2005) A PCR-based molecular marker applicable for marker-assisted selection for anthracnose disease resisatnce in lupin breeding. Cell Mol Biol Lett 10:123-134

Yu G-X, Wise RP (2000) An anchored AFLP- and retrotransposon-based map of diploid *Avena*. Genome 54:484-497

Yu J-K, Kantety RV, Graznak E, Benscher D, Tefera H, Sorrells ME (2006) A genetic linkage map for tef [*Eragrostis tef* (Zucc.) Trotter] Theor Appl Genet 113:1093-1102

Yue B, Cai X, Vick BA, Hu J (2009) Genetic diversity and relatiohsips among 177 public sunflower inbred lines assessed by TRAP markers. Crop Sci 49:1242-1249

Zhang F, Chen S, Chen F, Fang W, Chen Y, Li F (2011) SRAP-based mapping and QTL detection for inflorescence-related traits in chrysanthemum (*Dendranthema morifolium*). Mol Breeding 27:11-23

Zhang JJ, Shu QY, Liu ZA, Ren HX, Wang LS, De Keyser E (2012) Two EST-derived marker systems for cultivar identification in tree peony. Plant Cell Rep 31:299-310

Zhang W-K, Wang Y-J, Luo G-Z, Zhang J-S, He C-Y, Wu X-L, Gai J-Y, Chen S-Y (2004) QTL mapping of ten agronomic traits on the soybean (*Glycine max* L. Merr.) genetic map and their association with EST markers. Theor Appl Genet 108:1131-1139

Zhao W, Fang R, Pan Y, Yang Y, Chung J-W, Chung I-M, Park Y-J (2009) Analysis of genetic relationships of mulberry (*Morus* L.) germplasm using sequence-related amplified polymorphism (SRAP) markers. Afr J Biotech 8:2604-2610

Zheng BS, Yang L, Zhang WP, Mao CZ, Wu YR, Yi KK, Liu FY, Wu P (2003) Mapping QTLs and candidate genes for rice root traits under different water-supply conditions and comparative analysis across three populations. Theor Appl Genet 107:1505-1515

Zhuang Y, Zhou X, Wang S (2012) Genetic diversity of NBS-LRR class disease-resistance gene analogs in cultivated and wild eggplants. Plant Syst Evol 198:1399-1406

Zou J, Fu D, Gong H, Qian W, Xia W, Pires JC, Li RY, Long Y, Mason AS, Yang T-J, Lim YP, Park BS, Meng J (2011) De novo genetic variation associated with retrotransposon activation, genomic rearrangments and trait variation in recombinant inbred line population of *Brassica napus* derived from interspecific hybridization with *Brassica rapa*. Plant J 68:212-224.
